# Photocurable Oil-Based Thermosets Containing Modifiers from Renewable Sources for Coating Applications

**DOI:** 10.1021/acspolymersau.4c00068

**Published:** 2024-10-28

**Authors:** Vojtěch Jašek, Jan Fučík, Otakar Bartoš, Silvestr Figalla, Radek Přikryl

**Affiliations:** † Institute of Materials Chemistry, Faculty of Chemistry, Brno University of Technology, 61200 Brno, Czech Republic; ‡ Institute of Environmental Chemistry, Faculty of Chemistry, Brno University of Technology, 612 00 Brno, Czech Republic

**Keywords:** oil-based coating, reactive diluent, polarity modifier, paper coating, dip coating, metal coating, wood coating

## Abstract

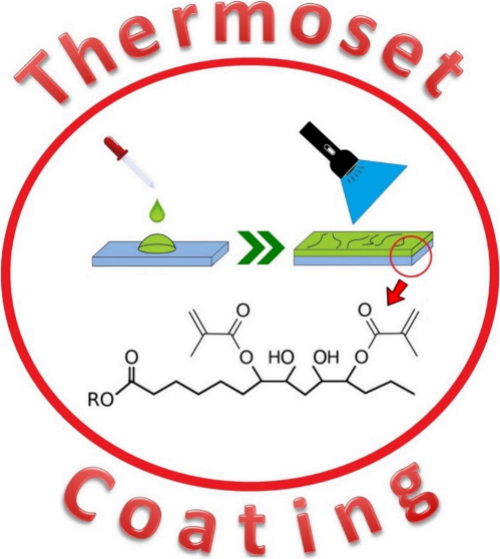

Coating materials involving nature-inspired compounds or renewable sources have recently attracted vast attention. This article presents the synthesis of modified rapeseed oil (MRO) as a precursor possessing high biobased carbon content suitable for cured thermosets formation. Two reactive diluents based on renewable sources, methacrylated methyl 3-hydroxybutyrate (M3HBMMA) and ethyl 3-hydroxybutyrate (E3HBMMA), were successfully synthesized. Lastly, isosorbide monomethacrylate (MISD) was suggested and produced as a polarity modifier miscible with modified curable oil systems capable of increasing the thermoset surface energy. All synthesized compounds were structurally analyzed via NMR, ESI-MS, and FTIR. The characterized reactive substances were coated on paper, stainless steel, and beech wood to investigate their suitability for forming thin layers. The paper dip coating verified the reactive diluting properties of M3HBMMA, resulting in the average formed coating deviation decrease (87.5% for undiluted MRO and 28.0% for 50 wt % M3HBMMA containing MRO). Also, the additional cured thermoset weight decreased from 350 to 69 wt % for the same systems. The standardized bend test applied on the coated stainless steel specimens revealed the thermoset’s flexibility and adhesion increase from a 12 ± 2° bending angle of 100% pure MRO to a 121 ± 2° bending angle measured for 40 wt % E3HBMMA containing the MRO-based thermoset. The coated beech wood samples underwent the standardized cross-hatch test investigating the substrate’s coating quality. The 100% MRO reached a level 1 rating (second worst), while the system with 40 wt % of MISD obtained a level 5 rating (the best).

## 1 Introduction

Curable vegetable oil-based precursors are attractive, available, and highly studied materials based on renewable sources, seeking their utility in various application fields.^1−3^ The vast amount of attention toward this thermoset-forming group of compounds attracts their high carbon content obtainable from natural sources, which can improve eventual CO_2_ emissions calculation due to its addition to conventionally petroleum-based systems.^4,5^ Triacylglycerols containing unsaturated fatty acids [monounsaturated fatty acids (MUFA) and polyunsaturated fatty acids (PUFA)] can be effectively transformed into materials capable of being modified to reactive systems suitable for material purposes.^6^ Product carbon footprint (PCF) calculation is conventionally performed according to ISO 14067. The usage of modified vegetable oils favors the PCF-calculated values since the carbon utilized in the oil’s structure is interpreted negatively in the summarization due to the plant’s CO_2_ utilization. Therefore, the PCF levels referred to by the supplier and the producer of oil-based curable systems can improve the eventual CO_2_ emission levels.^47^ Unsaturated double bonds undergo epoxidization, forming epoxy functional group formation within the oil carbon backbone.^7−9^ The most described, studied, and published are epoxidization mixtures containing hydrogen peroxide (H_2_O_2_) reacting with specific monocarboxylic acid (formic^10,11^ and acetic^12,13^) to form percarboxylic acid, which reacts with double bonds producing the epoxy groups. This reaction was previously described in numerous articles.^10−13^ In our previous paper, we included the process of residual peroxide removal via potassium iodide (KI) and sodium thiosulfate (Na_2_S_2_O_3_) to limit the presence of free peroxide in the epoxidized oil.^14^ These species can complicate further oil transformations to reactive precursor compounds capable of radical polymerization.^15^ Such systems are typically synthesized from acrylic,^16^ methacrylic,^17^ or itaconic acid.^18^ Also, the respective anhydrides of named acyl donors are used to modify epoxidized oils.^19,20^ Modified reactive vegetable oils are perspective materials for SLA 3D printing,^2,3,17^ pultrusion,^21,22^ or multicomponent coatings.^1^ Light-emitting diodes (LEDs) play a critical role in their availability and functionality. This irradiation source was introduced in 2002 and gained massive popularity due to numerous aspects: low energy consumption, low cost for production, minor heat generation during the emitting, or the countless possible wavelengths reaching hardware constructions assured by particular doped components. LEDs are usually used in 3D printers or other instrumentation for thermoset fabrication and ensure adequate and available progress in photocurable materials research.^46^

The organic and polymeric layers applied on metal substrates are a big group of precursor primers formulated for the coating industry.^23^ Flexible, transparent, and inert surface treatment mainly serves as an anticorrosive protection for these substrates.^24−26^ Several devices and products are exhibited in highly wet surroundings, often containing various salts, acidic, or basic components, which leads to the metal reactivity with the enriched water solution, resulting in the devices’ surface changes and damages.^23^ Apart from the reactive acrylic and methacrylic curable vegetable oils, many other material types are incorporated into such systems. Alkyds (polyester resins modified by additional acids or fatty acids) are often used for these purposes, usually in combination with specific rheological modifiers such as styrene, vinyl toluene, methyl methacrylate, butyl acrylate, or acrylic/methacrylic acid.^23,27^ These resins are commercially used, carrying particular market names such as CHS-ALKYD.^28^ Due to the higher flexibility demands, the oil-urethane systems are also suitable for metal-coating applications.^29,30^ Certain polyurethanes containing long-chain polyols and poly(methylene diphenyl diisocyanate) (PMDI) can be enriched with biobased content when various oils are incorporated. Particularly, mono- and diacylglycerides containing free hydroxyl groups, similar to pure castor oil, are added into the reactive polyurethane systems (as a part of the polyol component, which is eventually mixed with MDI).^29,30^ In particular, the combined acrylic-urethane coatings combining high *T*_g_ acrylic polyol (interpreted with the *T*_g_ value of 75 °C) served as multifunctional layers exhibiting antigraffiti, anticontamination, and antiadhesion properties.^29^ According to the literature, the acrylic and methacrylic radically polymerized coatings (containing certain reactive or functional additives such as amino acid compounds^24^ or Henna leaves extract^25^) were described as exceptional anticorrosion treatments.

Generally, the coatings applied to the polar substrates require specific component selection to ensure the prepared products’ ideal adhesion, compatibility, and functionality.^31,32^ The curable and organic coatings used for the paper coating must exhibit certain viscosity levels for the optimal performable coating technologies (such as dip coating).^33^ The standard curable coating composition consists of multiple components: the main high-viscous matrix, usually representing 70 wt % of the system; the reactive diluents for the viscosity modification (∼25 wt %); photoinitiator represents up to 5 wt % in the system; eventually, the low-concentrated additives are present in such systems (surfactants, light stabilizer, and colorants).^34^ Apart from the rheological modification, the coatings applied on wood substrates usually require other specific properties, such as sufficient mechanical properties, barrier properties, and compatibility with the polar-based surface.^35,36^ The acrylated and methacrylated curable oils contain free hydroxyl groups within the carbon backbone structure (due to the nucleophilic substitution of carboxylic acid on the epoxy groups).^16,37^ However, the overall surface tension and surface energy of cured thermosets are insufficient for optical compatibility with wood substrates. Specific components ensure the oil-based layers’ polarity increase, namely various silicones,^35^ or solid phase polymers, and multicomponent systems such as nanocrystalline cellulose, liquefied wood dust, or liquefied pine sawdust.^36^

Polyurethanes are the mainly used thermoset systems for coating various substrates such as metals or woods.^53−55^ The application of PU coatings relies on the chemical principle of mixing polyols and isocyanates. Then, they are applied on the particular surface. Then, the catalysts (mainly amines) are added, and a thermoset is formed. The toxicity and nonsustainable character of used isocyanates are the main adverse effects of PU coatings.^53−55^ The thermoplastic coatings are also often used in numerous applications. Poly(lactic-*co*-glycolic) acid (PLGA) or poly(vinyl acetate) (PVAc) are often used for coating applications^56^ and exhibit a sustainable potential compared to commercially used styrene–butadiene polymers.^57^ However, the thermoplastic systems required additional reactive diluents for coating processing. Therefore, the VOC has a major adverse effect on these systems from a sustainability and economic viewpoint.^56,57,61^ The idea formulated and suggested in this work is to incorporate a reactive diluent into the coating. The reactivity of curable precursors ensures the incorporation of the diluent into the formed coat, which minimizes VOC in the product. The application of biobased structures also enhances the sustainable character of such formed products.^59,60^

This paper is focused on the coating system formulation based on the methacrylated rapeseed oil (MRO) as a main component, and liquid curable additives, methacrylated methyl 3-hydroxybutyrate (M3HBMMA), methacrylated ethyl 3-hydroxybutyrate (E3HBMMA), and isosorbide monomethacrylate (MISD), modifying the layer’s rheology and polarity. The syntheses of all named components are described in our previously published articles.^14,38,39^ The methacrylated oils, alkyl carboxylates, or modified isosorbide can be used for various different applications beyond the coatings, such as stereolithography 3D printing,^42^ waterborne adhesives,^50^ or thermoset-based pultrusion.^58^ The other material utilities benefit mainly from the mentioned compounds’ biobased content and availability. This work directly focuses on applying suggested, synthesized, and characterized curable systems suitable for coating purposes. Various substrates were selected for the coating experiments: paper, stainless steel, and beech wood. All chosen materials were coated and characterized, and the applied layers were tested from a coating-forming capability standpoint. The dip coating and transparent layer-assisted coating were the experimentally investigated approaches. This paper’s innovative aspect lies in using our previously novel-synthesized less viscous methacrylated alkyl 3-hydroxycarboxylates, which have not yet been used for coating applications. Also, isosorbide monomethacrylate’s upgraded production was described previously, and the coating application is the novel proposed utility for this reactive compound.

## 2 Experimental Section

### 2.1 Materials

Epoxidized rapeseed oil was produced in our previously published paper^14^ from virgin rapeseed oil obtained from FICHEMA Inc. (Czech Republic). Despite the complete synthesis of alkyl carboxylates described in our recent paper,^38^ we used methyl and ethyl 3-hydroxybutyrates purchased at Sigma-Aldrich. Isosorbide used for isosorbide monomethacrylate synthesis was obtained from Novaphene, India. Other reactants, methacrylic anhydride (MAAH, 94%), methacrylic acid (MAA, 98%), catalysts potassium carbonate (K_2_CO_3_, 99%), triethylamine (TEA, 99%), photoinitiator phenylbis(2,4,6-trimethylbenzoyl)phosphine oxide (BAPO, 97%), solvent ethyl acetate (EtAc, 99%), and neutralization base sodium hydroxide (NaOH, 99%), were purchased at Sigma-Aldrich. The cellulose paper, stainless steel, and beech wood were all obtained from BAUHAUS, Czech Republic (local construction producer).

### 2.2 Structural Verification Methods

Nuclear magnetic resonance (NMR) was used to obtain the ^1^H and ^13^C spectra for structural confirmation. Bruker Avance III 500 MHz (Bruker, Billerica, MA, USA) with a measuring frequency of 500 MHz for ^1^H NMR and 126 MHz for ^13^C NMR was used for the measurements at the temperature of 30 °C using d-chloroform (CDCl_3_) as a solvent. Tetramethylsilane (TMS) served as an internal standard. The chemical shifts (δ) are expressed in part per million (ppm) units referenced by a solvent. Coupling constant *J* has (Hz) unit with coupling expressed as s—singlet, d—doublet, t—triplet, q—quartet, p—quintet, m—multiplet.

The molecular structure was also confirmed by mass spectrometry (MS) (Bruker EVOQ LC-TQ) using electrospray ionization (ESI). Product scan spectra were obtained by fragmentation of the following [M + H]^+^ precursor ions for all synthesized molecules (MRO, M3HBMMA, E3HBMMA, and MISD). Collision energy spread (5–20 eV) improved the collected MS/MS data quality. The obtained mass spectra were compared to their in silico prediction by CFM-ID 4.0,^40^ which also proposed the product ion structure for the most intensive masses.

Fourier-transform infrared spectroscopy (FTIR) served as an additional structure-verification method. The infrared spectrometer [Bruker Tensor 27 (Billerica, MA, USA)] was used, and the attenuated total reflectance (ATR) method was applied, where a diamond served as a dispersion component. The diode laser served as the irradiation source. A Michelson interferometer eventually quantified the measured signal. All illustrated spectra were composed of 32 total scans with a resolution of 2 cm^–1^.

Differential scanning calorimetry (DSC) confirmed the curability of the products. Also, this technique served for the comparison of compounds’ reactivity—the lower the peak temperature (*T*_p_) is, the more reactive the system is. The compounds (MRO, M3HBMMA, E3HBMMA, and MISD) were mixed with Luperox DI [*tert*-butyl peroxide, 1% (w/w) quantity added to the system]. The solutions were transferred to aluminum pans (5–7 mg) and sealed with the hermetic lid. The used instrument [DSC 2500 model from TA Instruments (New Castle, DE, USA)] served for the analysis. The sample exhibited continual heat increase (30–215 °C) with a 10 °C min^–1^ heating ramp.

Thermogravimetric analysis (TGA) determined every synthesized reactive precursor’s inflection points (*T*_max_). The samples used for TGA were the cured compounds analyzed by DSC previously. TGA performed on a TGA Q500 instrument [TA Instruments (New Castle, DE, USA)] illustrated the degradation process by the particular weight loss dependencies. The samples (5 mg) were measured under the following conditions: equilibration at 40 °C; heating to 600 °C at a heating rate of 10 °C/min under N_2_; heating to 650 °C at a heating rate of 10 °C/min under an air atmosphere.

The acidity value served as a method for determining the purity. Based on the reaction mechanics, methacrylic acid is the only secondary product in all syntheses. Therefore, determination of the acidity value prior to and after purification provides information regarding purity. The acidity value was measured according to ISO 660:2020.

The contact angle measurement investigated the hydrophobicity of the coated paper. We used a goniometer (the deviation ±1°) for the analysis, and See Software 7.0 was used for the surface energy quantification. The liquids used for the Lewis acid theory measurements were water, glycerol (polar), and di-iodo methane (nonpolar). Every coated paper sample (including 0–50 wt % M3HBMMA) underwent 10 measurements for every liquid. The final contact angle values were composed of the average values of all measurements. The surface energy levels were obtained from the software mentioned software.

### 2.3 Synthesis of Methacrylated Rapeseed Oil

**Scheme 1:**
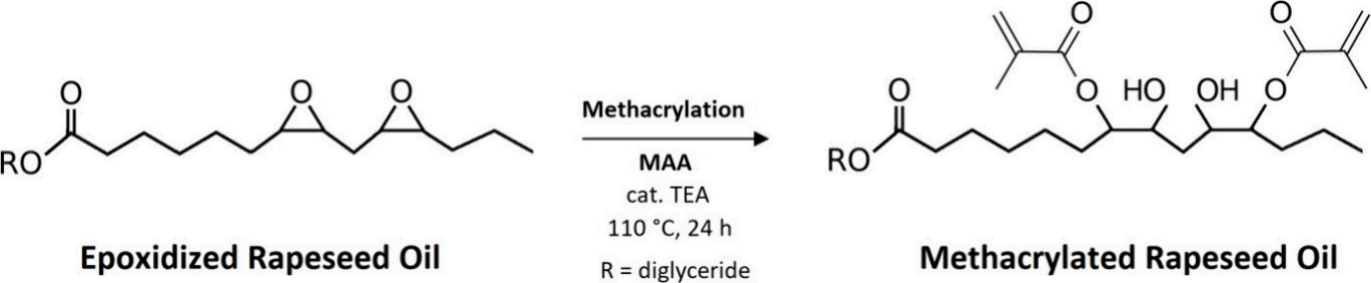
Methacrylation of Epoxidized Rapeseed Oil (ERO) Resulting in Methacrylated Rapeseed Oil (MRO) Production

The synthesis (Scheme 1) was described in detail in our previous article.^14^ The particular mass amount of epoxidized rapeseed oil (ERO) (calculated respectively to the OOC value, for the OOC reaching 6.1%, the total weight of ERO was 389 g) was mixed with MAA in the molar ratio (epoxy groups:MAA) = (1:1.5), which resulted in 195 g of MAA. This mixture was homogenized and set to 110 °C. Once the solution reached the reaction temperature, 2.6 wt % of catalyst (TEA, 15.6 g) was added to the mixture. The reaction lasted 24 h for the epoxy groups’ conversion maximized conversion. The eventual postreaction solution was mixed with EtAc (50 wt %) for the viscosity decrease, and the residual acidity was neutralized with NaOH in water solution (equimolar to the measured acidity). After neutralization, the mixture was washed with distilled water to remove the methacrylic salts. Eventually, the product was separated from EtAc via vacuum distillation. The obtained FTIR and ^1^H NMR spectral product confirmations are listed below (spectra are available in the Supporting Information):

^1^H NMR of methacrylated rapeseed oil (500 MHz, CDCl_3_): δ 6.16–6.09 (m, 2H), 5.58 (q, *J* = 2.1 Hz, 2H), 4.29 (dd, *J* = 11.9, 4.3 Hz, 2H), 4.18–4.08 (m, 3H), 2.30 (tt, *J* = 7.5, 2.3 Hz, 6H), 2.08–1.88 (m, 9H), 1.80–1.11 (m, 60H), 0.88 (tt, *J* = 7.1, 1.6 Hz, 7H).

FTIR spectrum absorption wavenumber intervals: O–H stretch of 3550–3200 cm^–1^, C–H stretch of (alkene) 3100–3000 cm^–1^, C–H stretch of 3000–2840 cm^–1^, C=O (ester) stretch of 1750–1735 cm^–1^, C=C stretch of 1662–1626 cm^–1^, C–O (ester) stretch of 1210–1163 cm^–1^, C=C bend of 840–790 cm^–1^.

### 2.4 Synthesis of Methacrylated Alkyl Carboxylates (M3HBMMA and E3HBMMA)

**Scheme 2:**
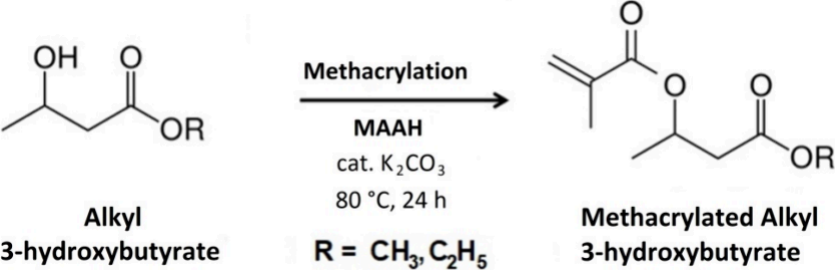
Methacrylation of Alkyl Carboxylates Producing Methacrylated Methyl and Ethyl 3-Hydroxybutyrate (M3HBMMA and E3HBMMA)

The syntheses producing methacrylated alkyl carboxylates (Scheme 2) were described, studied, and summarized in our published paper.^38^ The particular alkyl 3-hydroxybutyrate [methyl 3-hydroxybutyrate (M3HB), 300 g, or ethyl 3-hydroxybutyrate (E3HB) 300 g] was mixed with MAAH in an equimolar ratio (1:1) (392 g of MAAH for M3HB, and 350 g of MAAH for E3HB). The catalyst K_2_CO_3_ was added in a 1 wt % concentration (7 g into M3HB and 6.5 g into E3HB). The mixture was set to 80 °C, and the reaction was performed for 24 h. The reaction mechanics of methacrylation by methacrylic anhydride was investigated and published in our previous article.^48^ The catalyst was potassium acetate; however, the basicity is the main catalytic factor. Therefore, potassium acetate and potassium carbonate can achieve sufficient basic conditions. The eventual mix was neutralized similarly to the MRO described in the previous section (Section 2.3). The neutralized product was structurally analyzed by using FTIR, ESI-MS, ^1^H NMR, and ^13^C NMR:

#### 2.4.1 Methacrylated Methyl 3-Hydroxybutyrate (M3HBMMA)

^1^H NMR (CDCl_3_, 500 MHz): δ (ppm) 6.07–6.06 (dq; *J* = 1.96; 1.02; 0.98; 0.98 Hz; 1H), 5.56–5.53 (p; *J* = 1.60; 1.60; 1.58; 1.58 Hz; 1H), 5.35–5.29 (dp; *J* = 7.32; 6.26; 6.26; 6.25; 6.25 Hz; 1H), 3.68 (s; 3H), 2.72–2.67 (dd; *J* = 15.34; 7.29 Hz; 1H), 2.57–2.53 (dd; *J* = 15.35; 5.79 Hz; 1H), 1.92 (dd; *J* = 1.63; 1.01 Hz; 3H), 1.35–1.34 (d; *J* = 6.36 Hz; 3H).

^13^C NMR (CDCl_3_, 126 MHz): δ (ppm) 170.70; 166.59; 136.47; 125.41; 67.68; 51.73; 40.74; 19.89; 18.23.

ESI-MS analyzed the precursor [M + H]^+^ 187.1 *m*/*z*. Fragments: 155.1, 101.1, and 69.2 *m*/*z* according to CFM ID 4.0.

FTIR spectrum absorption wavenumber intervals: C–H stretch. 3000–2840 cm^–1^, C=O (ester) stretch. 1750–1735 cm^–1^, C=C stretch. 1662–1626 cm^–1^, C–O (ester) stretch. 1210–1163 cm^–1^, C=C bend. 840–790 cm^–1^.

#### 2.4.2 Methacrylated Ethyl 3-Hydroxybutyrate (E3HBMMA)

^1^H NMR (CDCl_3_, 500 MHz): δ (ppm) 6.07–6.06 (dd; *J* = 1.75; 0.97 Hz; 1H), 5.54–5.53 (q; *J* = 1.63; 1.63; 1.63 Hz; 1H), 5.35–5.29 (dp; *J* = 7.50; 6.24; 6.24; 6.24; 6.24 Hz; 1H), 4.16–4.10 (qd; *J* = 7.11; 7.06; 7.06; 0.96 Hz; 2H), 2.69–2.65 (dd; *J* = 15.28; 7.42 Hz; 1H), 2.55–2.51 (dd; *J* = 15.29; 5.75 Hz; 1H), 1.94–1.91 (m; 3H), 1.34–1.33 (d; *J* = 6.28 Hz; 3H), 1.25–1.22 (t; *J* = 7.13; 7.13 Hz; 3H).

^13^C NMR (CDCl_3_, 126 MHz): δ (ppm) 170.21; 166.54; 136.46; 125.33; 67.71; 60.57; 41.01; 19.85; 18.19; 14.14.

ESI-MS analyzed precursor [M + H]^+^ 201.0 *m*/*z*. Fragments: 156.0, 115.1, and 73.2 *m*/*z* according to the CFM ID 4.0.

FTIR spectrum absorption wavenumber intervals: C–H stretch. 3000–2840 cm^–1^, C=O (ester) stretch. 1750–1735 cm^–1^, C=C stretch. 1662–1626 cm^–1^, C–O (ester) stretch. 1210–1163 cm^–1^, C=C bend. 840–790 cm^–1^.

### 2.5 Synthesis of Isosorbide Monomethacrylate

**Scheme 3:**
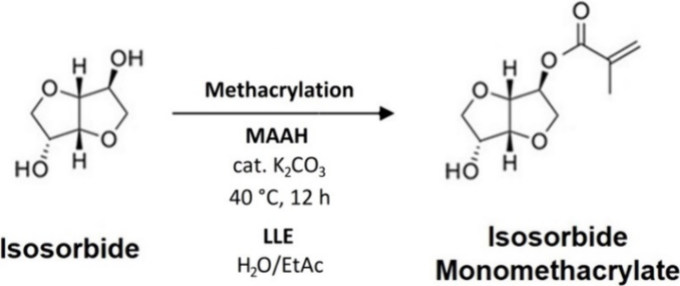
Partial Methacrylation of Isosorbide Resulting in Isosorbide Monomethacrylate (MISD) Production Involving the Purification by Liquid–liquid Extraction (LLE)

Isosorbide monomethacrylate synthesis (Scheme 3) was described, investigated, and experimentally verified in our previous article.^39^ The reaction catalysis mechanics is similar to M3HBMMA and E3HBMMA reactions.^48^ Isosorbide (1 mol, 146.2 g) was melted at 60 °C and mixed with catalyst K_2_CO_3_ (0.01 mol, 0.98 g). Once the reactant melted, MAAH (1 mol, 154.2 g) was transferred into a dripping funnel and continually mixed drop by drop into the melted isosorbide (this process lasted 4 h). After the MAAH quantitative addition, the temperature was decreased to 40 °C, and the reaction lasted an additional 8 h. After the reaction, the formed MAA was distilled from the product mixture. Liquid–liquid extraction (LLE) purification involves the addition of distilled water (300 g). The water phase containing MISD was extracted by EtAc (300 g) to transfer MISD into the EtAc solution. Lastly, MISD was separated from EtAc by vacuum distillation. This process was repeated three times to maximize the product yield. Pure MISD was characterized via FTIR, ESI-MS, and ^1^H NMR:

^1^H NMR (CDCl_3_, 500 MHz): δ (ppm) 6.17–6.11 (dt, *J* = 15.6, 1.3 Hz, 2H); 5.64–5.59 (dt, *J* = 3.1, 1.5 Hz, 2H); 5.24–5.18 (m, 1H); 4.92–4.88 (t, *J* = 5.1 Hz, 1H); 4.68–4.61 (t, *J* = 4.8 Hz, 1H); 4.57–4.49 (dd, *J* = 4.4, 1.2 Hz, 1H); 4.44–4.38 (dd, *J* = 4.6, 1.1 Hz, 1H); 4.37–3.33 (d, *J* = 3.2 Hz, 1H); 4.21–3.69 (m, 10H); 3.66–3.51 (dd, *J* = 9.5, 6.0 Hz, 1H); 2.68–2.51 (d, *J* = 7.0 Hz, 1H); 1.98–1.94 (m, 6H).

ESI-MS analyzed the precursor [M + H]^+^ 215.1 *m*/*z*. Fragments: 129.1 and 62.9 *m*/*z* according to CFM ID 4.0.

FTIR spectrum absorption wavenumber intervals: O–H stretch. 3550–3200 cm^–1^, C–H stretch. 3000–2840 cm^–1^, C=O (ester) stretch. 1750–1735 cm^–1^, C=C stretch. 1662–1626 cm^–1^, C–O (ester and alcohol) stretch. 1210–1163 cm^–1^, C=C bend. 840–790 cm^–1^.

### 2.6 Dip Coating With the Oil-Based Mixture

**Figure 1:**
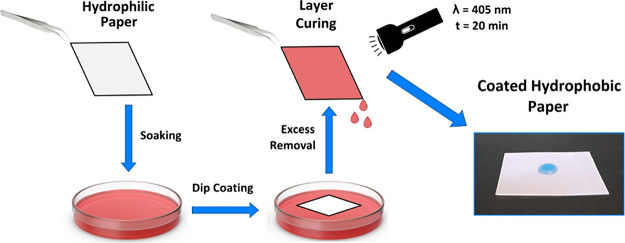
Performed dip coating using oil-based curable solution forming a hydrophobic paper.

The dip coating was applied to hydrophilic paper to verify the rheological modification of MRO using a low-viscosity reactive diluent, M3HBMMA, resulting in the hydrophobic coated paper formation. The coating process consisted of the following steps. The particular coating solutions were prepared (0, 10, 20, 30, 40, and 50 wt % of M3HBMMA in MRO), and 1 wt % of photoinitiator BAPO was added. The complete solution was placed into the Petri dish, and a preweighted piece of paper was dipped into the solution for 10 s. The coated paper was removed from the curable mixture, and the excess precursor solution was separated for 1 min. Subsequently, the coated paper was exhibited to the blue irradiation of LED (8 mW/cm^2^ of irradiation power, 405 nm wavelength) for 20 min. The formed layer thickness was measured; the added polymer weight was analyzed, and the hydrophobic character after the coating was confirmed. The scheme of performed dip coating is displayed in Figure 1.

### 2.7 Transparent Layer-Assisted Coating by the Oil-Based Mixture

**Figure 2:**
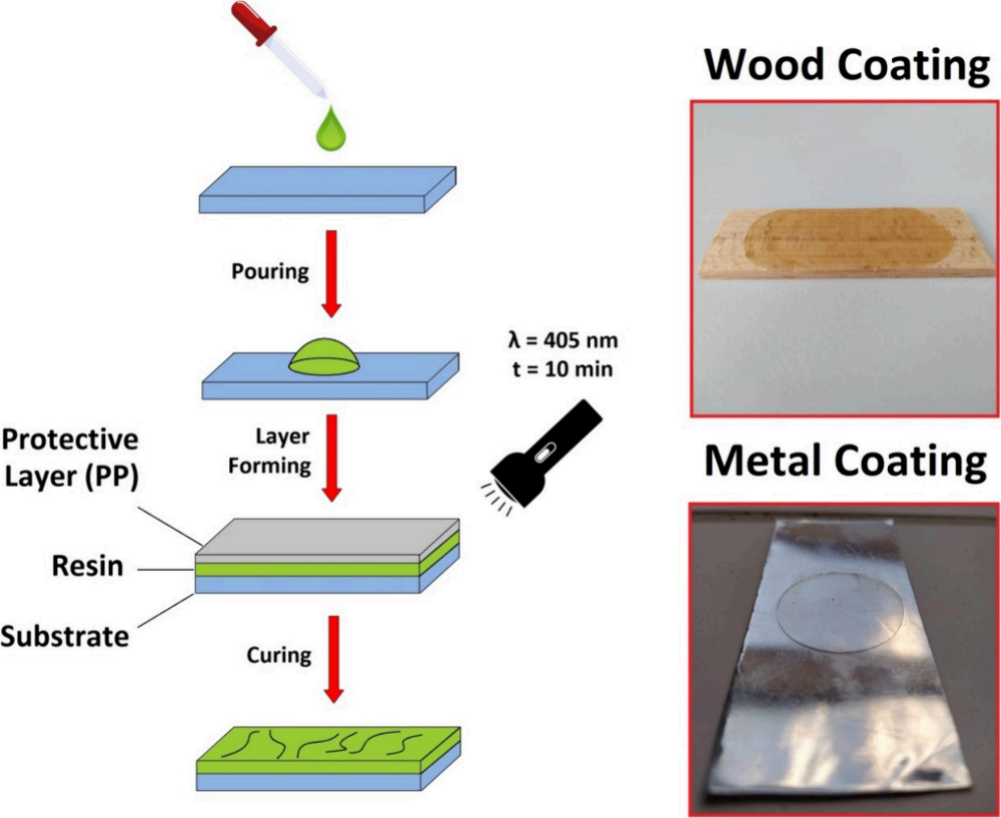
Performed transparent layer-assisted coating of stainless steel and beech wood by an oil-based curable coating system.

The metal and wood were coated by an oil-based precursor solution via a transparent layer-assisted approach, as schematically displayed in Figure 2. This procedure was chosen due to the laboratory scale of experiments. The rotating cylinders are an up-scaled alternative to this process. In this article, we aimed for the proposed presentation of the rheological, mechanical, and chemical properties of the used materials. The metal was coated by the oil solution containing E3HBMMA (0, 10, 20, 30, and 40 wt %) due to E3HBMMA’s flexibility character, assuring better adhesion and mechanical properties of the eventual product. The beech wood was treated by the curable system involving MISD (0, 10, 20, 30, and 40 wt %) to study the wood-thermoset adhesion enhancement and increased resistance against mechanical damage. The prepared MRO-based curable mixture was poured on the substrate’s surface, covered by the transparent (PP) layer, set to a defined layer thickness, and cured by an LED irradiation source (8 mW/cm^2^ of irradiation power, 405 nm wavelength) for 10 min. The polymer cover on the metal substrate was analyzed using applied norm ASTM D522. The polymer coating on the beech wood was investigated via the cross-hatch method, ISO 2409.

## 3 Results and Discussion

### 3.1 Oil-Based Coating Component Characterization

**Figure 3:**
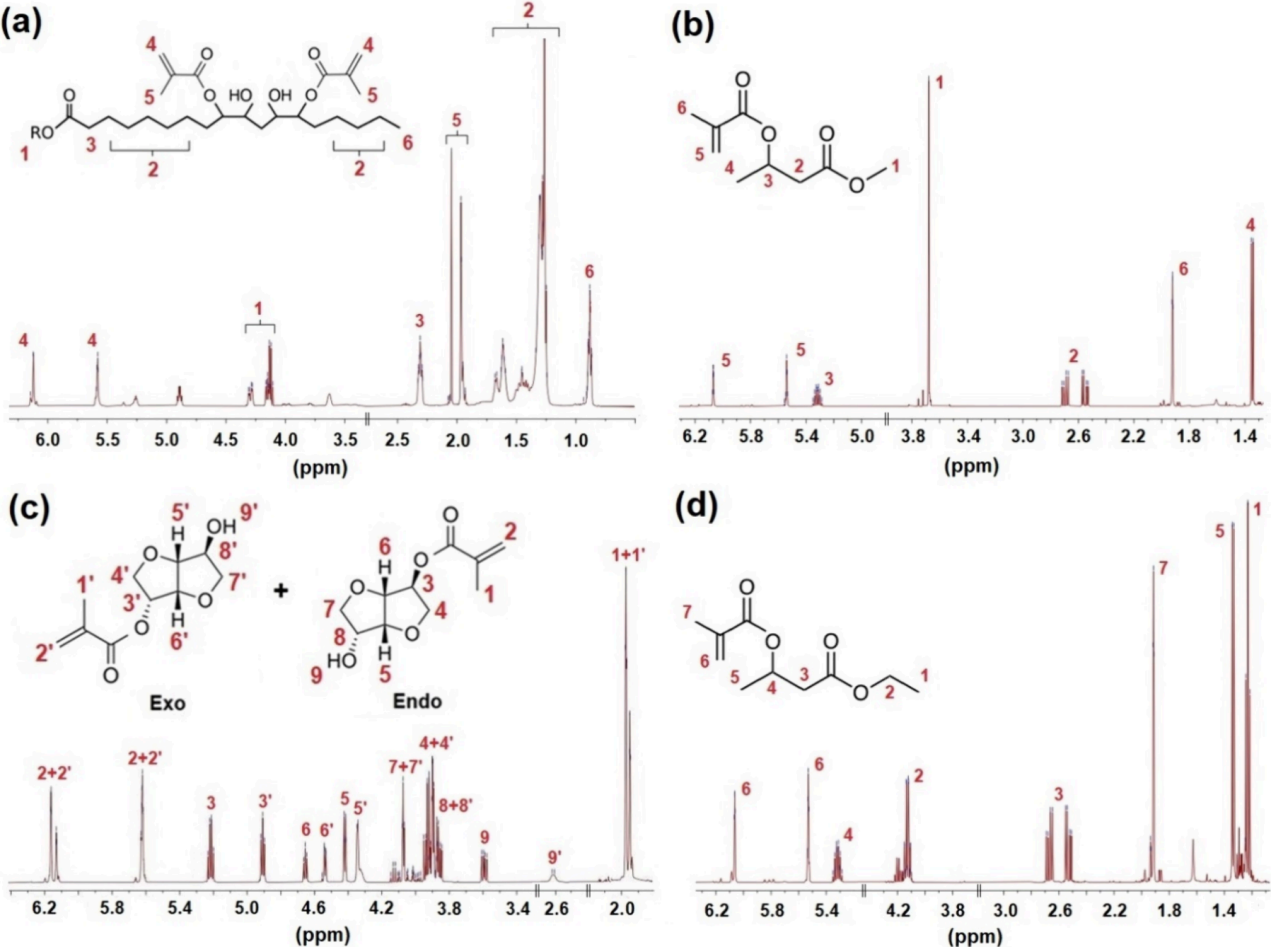
Obtained ^1^H NMR spectra of all synthesized curable compounds used for the coating applications. (a) Methacrylated rapeseed oil (MRO), (b) methacrylated methyl 3-hydroxybutyrate (M3HBMMA), (c) isosorbide monomethacrylate (MISD), and (d) ethyl 3-hydroxybutyrate (E3HBMMA). Reproduced from ref (38). Available under the CC-BY-4.0 license. 2022 by Vojtch Jaek et al.

The synthesized products were characterized via numerous structure-verification methods, and the results and spectra obtained are given in the Supporting Information. The ^1^H NMR spectra of all obtained compounds [methacrylated rapeseed oil (a), methacrylated methyl 3-hydroxybutyrate (b), isosorbide monomethacrylate (c), and ethyl 3-hydroxybutyrate (d)] are shown in Figure 3 to present successful syntheses. The chosen reaction conditions for methacrylation of M3HBMMA, E3HBMMA, and MISD are based on the results from our previous publications.^38,39,48^ It was found that MISD’s conversion reached above 90% conversion after 10 h^39,48^; the conversions of M3HBMMA and E3HBMMA were monitored in time (59.5% M3HBMMA and 45.6% E3HBMMA).^38^ Based on these results, the synthesis was set to 24 h to achieve the maximal conversion. The chosen reaction temperatures were selected from between 60 and 80 °C, and most of the reactions achieved high yields in a published review focused on the DMAP-catalyzed methacrylation.^49^ The catalyst’s load was set to 1 mol % in our work. However, the catalyst concentration was much higher in previously published articles (10 mol %), while the reaction time lasted 16–24 h.^50^ Based on these published results, we chose 80 °C and 24 h reaction time for M3HBMMA and E3HBMMA and 12 h for MISD.

The ^1^H NMR spectra of M3HBMMA (Figure 3b) and E3HBMMA (Figure 3d) contain minimum undefined signals (E3HBMMA contains only negligible peaks at 3.7 and 1.6 ppm), which are probably minor impurities from the methacrylation (either residual MAA of MAAH). The spectrum of MRO (Figure 3a) contains wider regions of connected multiplet peaks. This results from an undefined triacylglyceride complex structure containing several fatty acids. As a result, these particular signals form wide shift intervals, which are integrated (displayed in the Supporting Information). The essential signals lie in shifts 5.6 and 6.1 ppm, confirming the presence of unsaturated double bonds of methacrylate functional groups. The ^1^H NMR spectrum of MISD (Figure 3c) reveals the racemic mixture of exo and endo monomethacrylates. This is a consequence of nonselective chemical synthesis using anhydride as an acyl donor. However, all the signals in the spectrum confirm the structure of the monomethacrylated isosorbide. All presented spectral numerical values were previously published in our articles on synthesis kinetics, catalysis, and product isolation.^14,38,39^

**Figure 4:**
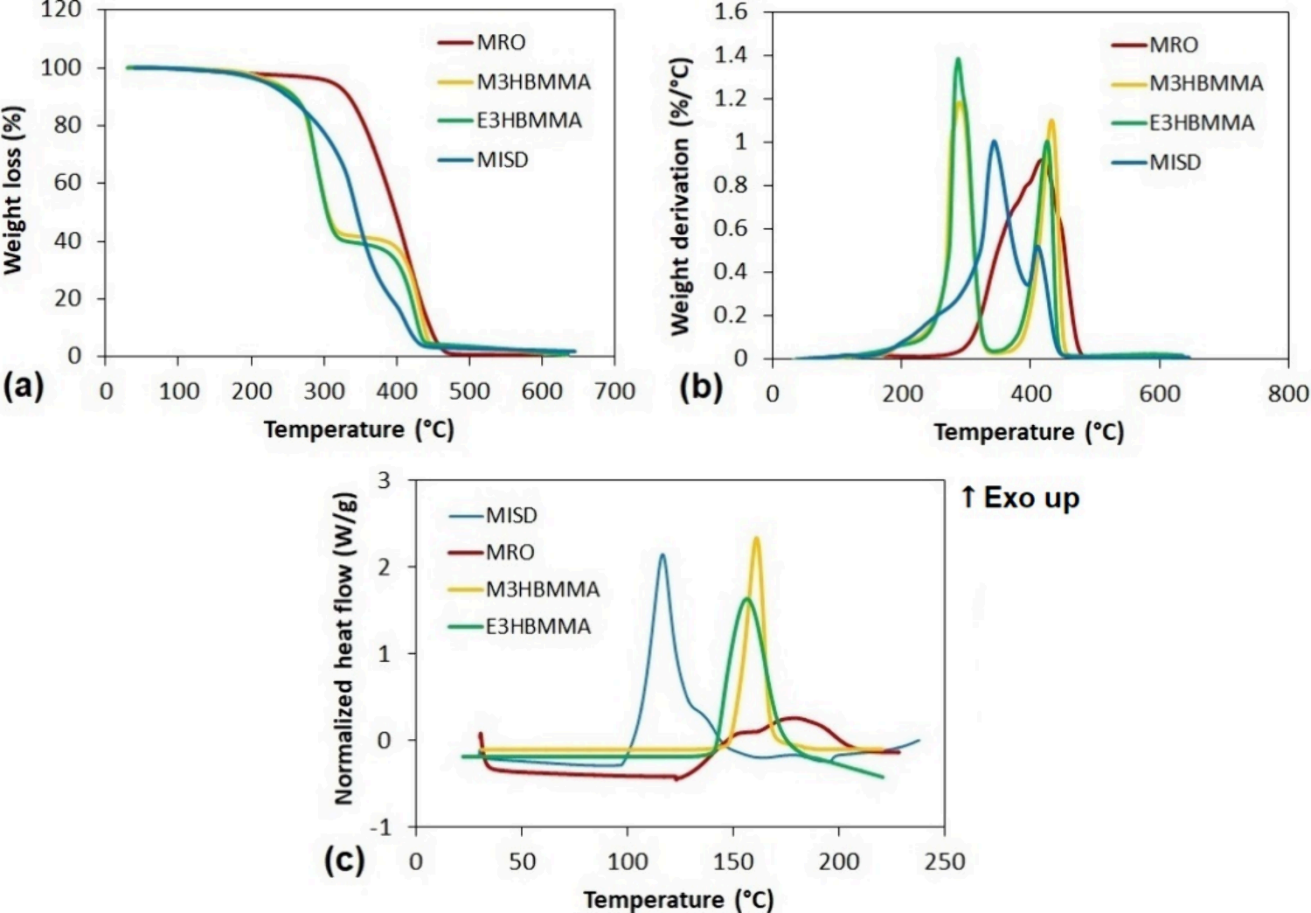
Thermal analysis of synthesized products. (a) TGA integral results, (b) TGA derivative results, and (c) DSC curability study.

All products were also characterized via thermal analyses (TGA and DSC) to report their additional properties. The results are shown in Figure 4 [TGA results in (a) and (b); DSC illustrated in (c)]. The differences are evident from both analyses. TGA uncovered the exceptional thermal stability of MRO due to its complex carbon structure (*T*_max_ reaching 417.5 °C) compared to MISD reaching a lower thermal stability (*T*_max_ 343.2 °C). The lowest thermal stability exhibits both M3HBMMA and E3HBMMA (*T*_max_ around 285 °C) due to ester bonding in their structure. The performed DSC uncovered the specific difference between MRO and other compounds since the measured exothermic peak reached a much lower maximum value compared to M3HBMMA, E3HBMMA, and MISD but formed a much wider signal. This outcome signalizes lower and longer-lasting reactivity of MRO. The other three systems exhibited narrow, high peaks, signalizing exceptional and quantitative curability. The comparison of MISD with methacrylated alkyl carboxylates uncovers a higher MISD reactivity due to the lower *T*_p_ value (117.1 °C) than M3HBMAA and E3HBMMA (*T*_p_ reaching 161.0 and 156.7 °C, respectively). The combined results from TGA and DSC show a positive impact on final cured thermoset properties since MRO enhances the thermal stability and MISD, M3HBMMA, and E3HBMMA increase the reactivity of the precursor system. All measured values and results are summarized in Table 1. The summarization involves the gravimetric yields determined by the weight measurement compared with the theoretical calculation. Also, the products’ purity is present in Table 1, which is determined by the residual acidity value compared to the initial value prior to methacrylic acid isolation. Lastly, the biobased weight content is included in the summarized results. The values were determined according to their structure and meticulously analyzed via NMR, ESI-MS, and FTIR. The importance of biobased content determination is detailed and described in the published literature.^51^

**Table 1:** Summarized Results of Thermal Analyses and Syntheses

system	DSC peak temperature *T*_p_ (°C)	TGA inflection point *T*_max_ (°C)	bio-based mass content (%)	product yield (gravimetrically) (%)	purity (acid value) (%)
MRO	179.1	417.5	76.62	94.5	98.9
M3HBMMA	161.0	284.8	55.85	98.3	99.8
E3HBMMA	156.7	285.5	74.93	98.0	99.9
MISD	117.1	343.2	68.30	61.8	99.5

### 3.2 Dip Coating of a Hydrophilic Paper

The paper is the fibrous cellulose substrate exhibiting a highly hydrophilic character due to the many unbonded free hydroxyl groups within the cellulose carbon backbone structure. This characteristic property leads to the paper’s modification using several polymeric or organic-based systems, assuring the paper’s wetting prevention.^41^ Apart from the structural characterization of the coated organic layer, the rheological, adhesive, and mechanical properties must be studied to perform the coating process successfully. The enhancement of MRO material properties by the methacrylated alkyl carboxylates was investigated in our published papers.^14,42^ The decreased apparent viscosity is evident from the results illustrated in Table 2. We used measured rheological characterization from our previous work. The particular weight contents of M3HBMMA are directly connected with the viscosity value in Table 2.^14^ SLA 3D printing was the leading application field in the published works. This article mainly focuses on the rheological and coating-applicable enhancements of alkyl carboxylates in MRO-based systems. The dependency of MRO apparent viscosity on the methacrylated alkyl carboxylate (M3HBMMA) is summarized in Table 2.

**Table 2:** Obtained Rheological Dependency of the Methacrylated Alkyl Carboxylate in Methacrylated Rapeseed Oil (MRO)^a^

MRO rheological modification	
*w*_M3HBMMA_ (wt %)	η (mPa s)
0	1790.2
5	1254.6
10	836.0
15	594.7
20	420.0
25	301.3
30	234.7
35	189.7
40	158.6
45	142.6

a Reproduced from ref (14). Available under the CC-BY-4.0 license. 2023 by Vojtch Jaek et al.

**Figure 5:**
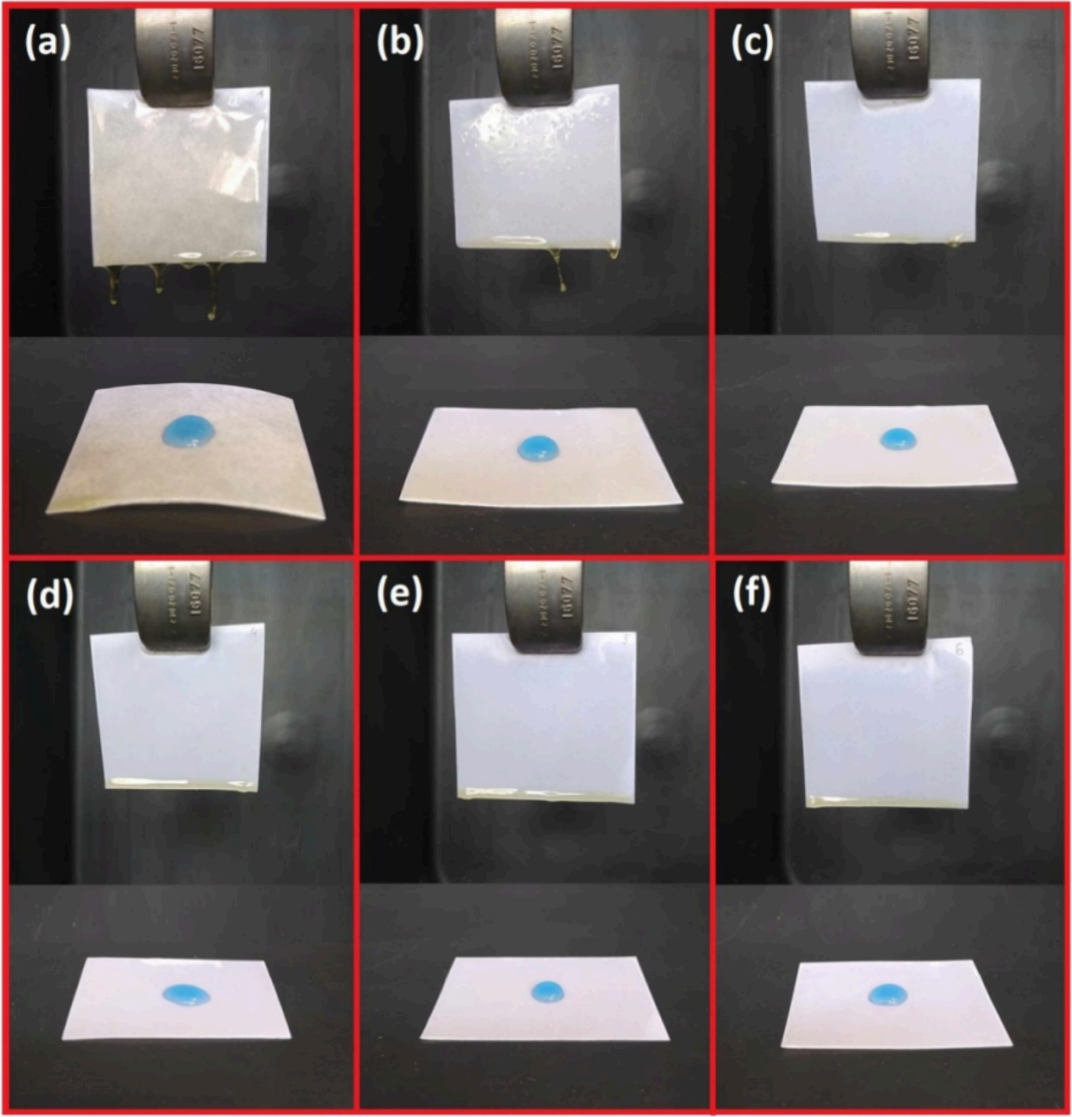
Performed paper-dip coating using an oil-based curable system involving the reactive diluent, methacrylated methyl 3-hydroxybutyrate (M3HBMMA), in varying weight concentrations. (a) 0% M3HBMMA, (b) 10% M3HBMMA, (c) 20% M3HBMMA, (d) 30% M3HBMMA, (e) 40% M3HBMMA, and (f) 50% M3HBMMA.

Based on our previous findings, we prepared the defined varying concentrations of M3HBMMA as a rheological modifier and reactive diluent in our synthesized MRO-based curable system. Methacrylated alkyl carboxylates exhibit specific low apparent viscosity (∼3 mPa s);^14^ therefore, they were selected as potential candidates for reactive diluting purposes. Also, we decided to use M3HBMMA for the paper coating (E3HBMMA was chosen for the metal coating) due to the molecules’ innovativeness in the coating utility field. Previously, methacrylated alkyl carboxylates were applied mainly for SLA 3D printing.^42^ The dip coating was performed with the prepared photoinitiator-containing curable system with the varying weight content of M3HBMMA, and the layer thickness was measured in different paper regions. Subsequently, the weight content increased by the thermoset surface treatment was analyzed via gravimetry. Eventually, the hydrophobic character assured by the polymer coating was confirmed for all prepared specimens. The dip coating performed using different curable systems is displayed in Figure 5. The paper coating measurement results are shown in Figure 7.

**Figure 6:**
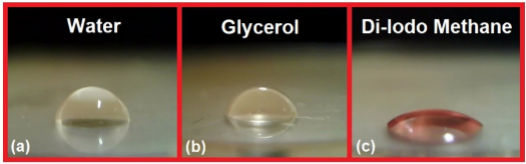
Performed contact angle measurements for 50 wt % of the M3HBMMA system. (a) Measurement with water, (b) measurement with glycerol, and (c) measurement with di-iodo methane.

**Figure 7:**
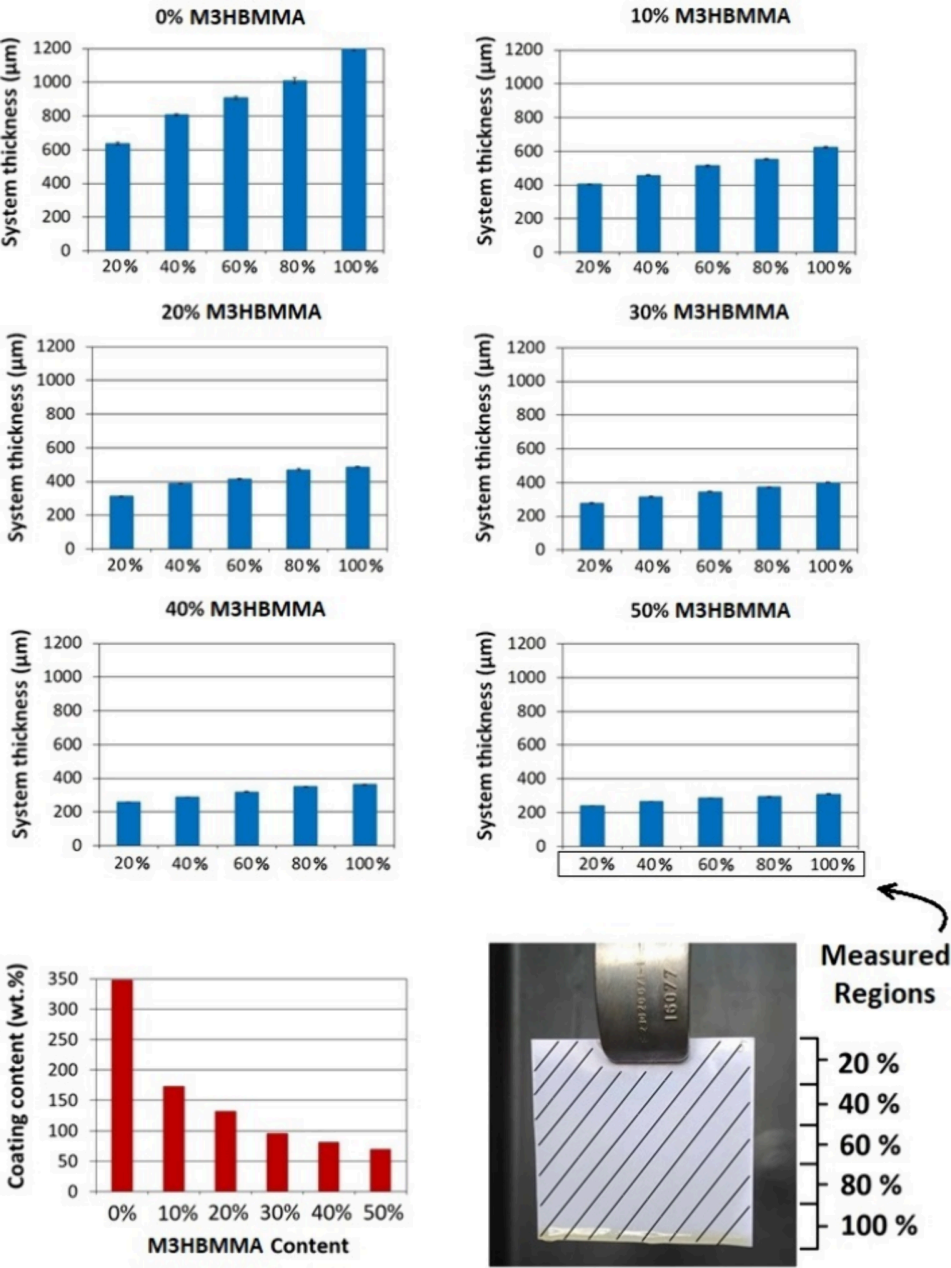
Paper dip coating analysis results involving the layer thickness measurement for the systems with varying layer thickness measured in the different coated-paper regions and the thermoset weight content gravimetric analysis.

The varying system viscosities and results of the coating technology process are evident in Figure 5. The pure MRO system (Figure 5a) contains cured drips on the bottom of the treated paper compared with the systems with higher reactive diluent content (Figure 5d–f). The rapid polymeric weight content is also observable from the demonstrated pictures since the coated paper with 0 wt % of M3HBMMA bent the paper due to most of the thermoset content. The insufficient viscosity decrease is detectable for the systems below 30 wt % of M3HBMMA (Figure 5a–c). This outcome confirms the reactive diluent requirements in other paper-coating articles in the published literature: the reactive diluent content was generally set to approximately 25 wt %^34^ or 30 wt % for particularly oil-coated paper.^41^ The measured coated layer thicknesses (Figure 7) also further investigated the formed coat homogeneity in the different measured paper regions. The 0 wt % M3HBMMA-coated system exhibited a layer thickness interval ranging from 640 to 1200 μm (87.5% spread across the coated area). On the other hand, the slightest thickness deviations exhibited the most diluted paper, reaching a 28.0% spread. The breaking concentration for the potential applications (30 wt % M3HBMMA content) reached 43.6% inconsistency across the coated substrate. These values can be decreased by selecting alternative coating techniques. The thermoset weight content on the coated paper varied from 350 wt % increase (0 wt % of M3HBMMA) to 69 wt % (50 wt % of M3HBMMA).

We measured the contact angle to quantify the surface energy value for all of the prepared coated papers. The results are summarized in Table 3. The demonstrated contact angle measurements using water, glycerol, and di-iodo methane are displayed in Figure 6. The results confirm that the hydrophobic character slightly increases with M3HBMMA content. This minor trend is caused by the free hydroxyl functional group minimization in the system with the rising M3HBMMA content. The hydroxyl groups are present in the MRO structure (see Scheme 1). However, the hydrophobic character is basically unchanged with the reactive diluent content. The repulsive character toward water is simplified in Figure 5. According to available sources, the uncoated paper exhibits exceptional hydrophilic character.^52^

**Table 3:** Summarized Results of Contact Angle Measurements

system	contact angle (Water)	contact angle (Di-Iodo Methan)	contact angle (Glycerol)	free surface energy γ_S_ (mJ/m^2^)
0% M3HBMMA	85° ± 3°	35° ± 3°	71° ± 3°	42.6
10% M3HBMMA	86° ± 2°	35° ± 2°	73° ± 1°	42.0
20% M3HBMMA	88° ± 3°	34° ± 2°	72° ± 1°	42.9
30% M3HBMMA	90° ± 3°	34° ± 3°	73° ± 2°	42.9
40% M3HBMMA	90° ± 1°	34° ± 1°	72° ± 3°	42.9
50% M3HBMMA	91° ± 3°	33° ± 3°	75° ± 3°	42.9

### 3.3 Transparent Layer-Assisted Metal Coating

**Figure 8:**
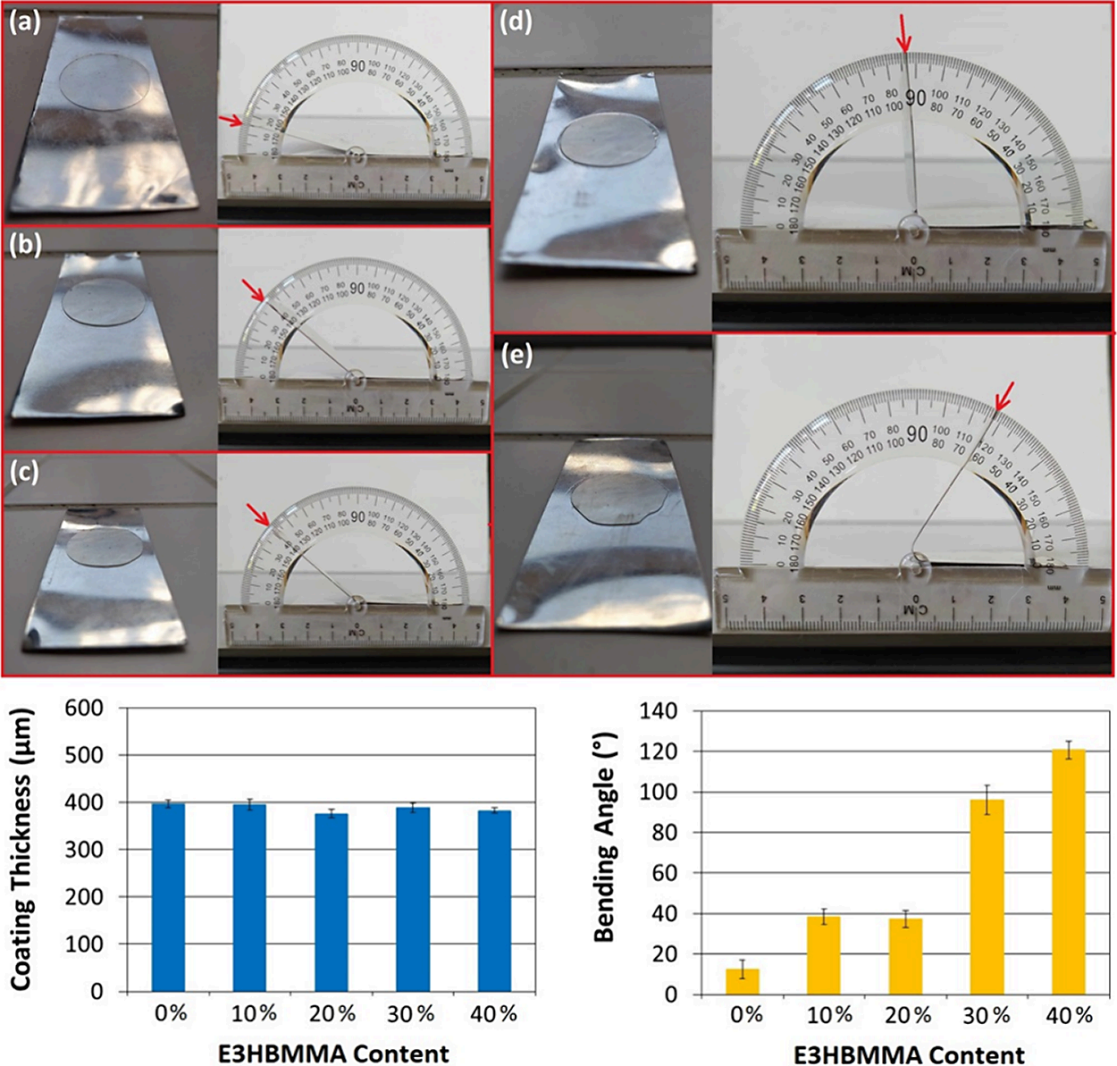
Formed polymer coating on stainless steel substrates, coating thicknesses, and bending angle measurement results. The experimental bending angle apparatus for MRO-based thermoset coatings. (a) 0 wt % E3HBMMA, (b) 10 wt % E3HBMMA, (c) 20 wt % E3HBMMA, (d) 30 wt % E3HBMMA, and (e) 40 wt % E3HBMMA.

The metal coating was formed from an MRO-based curable system containing E3HBMMA as a reactive diluent. According to the used norm for coating analysis and the industrial requirements for polymer coatings on metal surfaces, the adhesion and flexibility of the applied coat are the key parameters.^43^ Based on our previous findings, E3HBMMA is a suitable candidate for the MRO system reactive diluting since this curable monomer exhibits low *T*_g_ value (below 25 °C), convenient storage modulus (860 MPa at 28 °C), and relatively high flexibility.^42^ On the other hand, MRO reaches high cross-linking density (56.4 kmol/m^3^) and exceptional thermal stability (heat-resistant index reaching 170.3 °C).^14^ These factors, in combination, signify the E3HBMMA’s suitability for the conveniently applied flexible and thermally stable polymer coating on the stainless steel surface. The coating process was performed using curable systems with different E3HBMMA weight contents (from 0 up to 40 wt %), and following the used norm, the bending procedure was done. The applied polymer coat thickness consistency was measured, and the bending angle at the layer deformation was determined. The results of the experimental investigations are shown in Figure 8.

The average thickness of formed layers formed at all substrates containing five triplicates in total reached 388 ± 8 μm, which results in an approximate 2% relative deviation across all prepared and coated systems. This value is sufficient according to the used norm. The determined bending angles are summarized for all prepared specimens in Figure 8a–e. The system’s flexibility and durability increase continually and gradually based on the determined bending angles. The MRO system containing 0 wt % E3HBMMA reached a bending angle of 12 ± 2°, while the most flexible system containing 40 wt % of E3HBMMA reached 121 ± 2°, signifying the significant flexibility and adhesion increase. The published metal bending tests were usually performed at a 15° bending angle.^43^ The trend did not progress between E3HBMMA concentrations of 10 and 20 wt % probably due to the standard measurement potential failed tests. However, the increasing flexibility and adhesion trend with the increasing E3HBMMA content was confirmed from the perspective of all prepared systems.

### 3.4 Transparent Layer-Assisted Wood Coating

**Figure 9:**
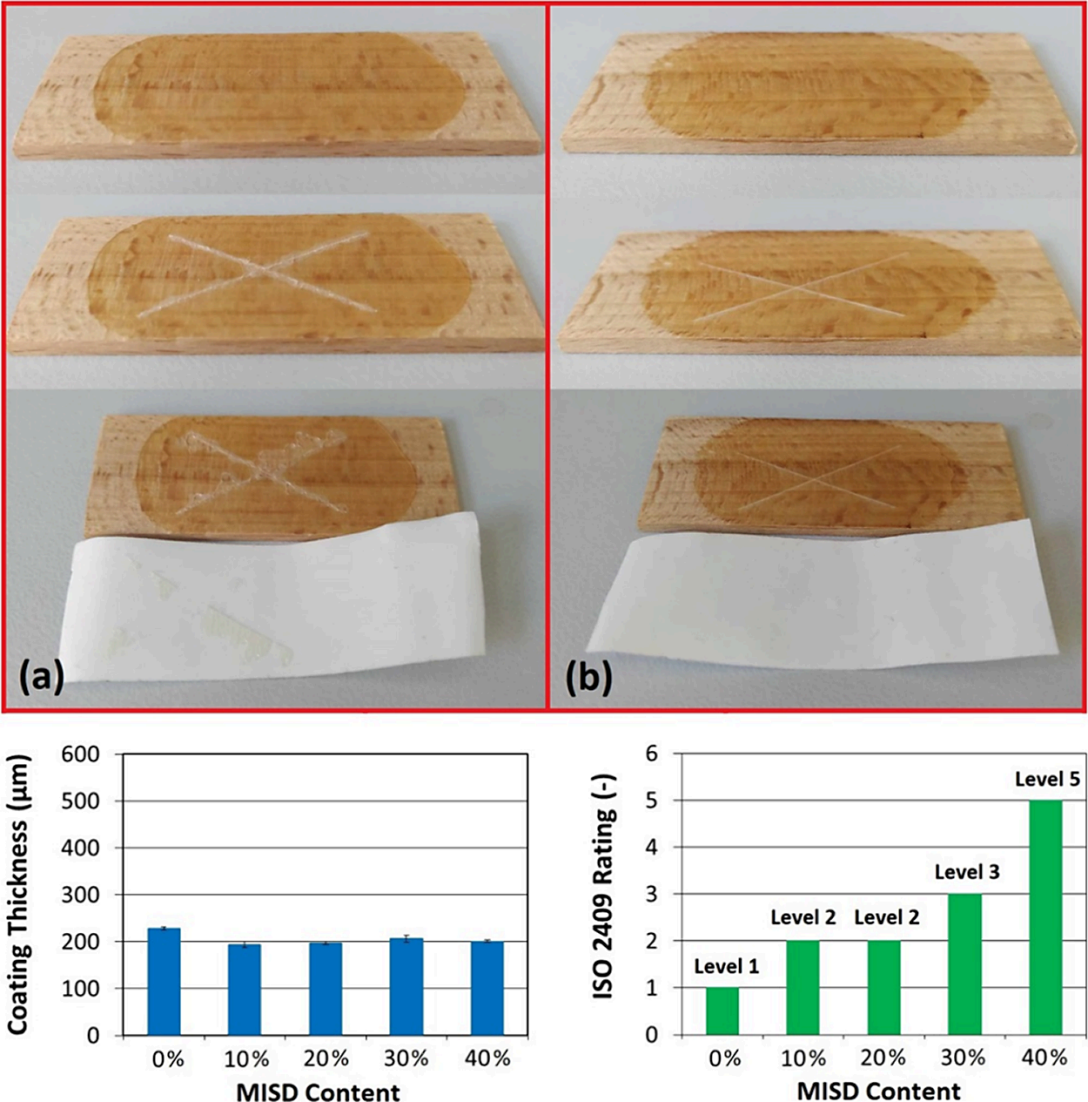
Wood coating results involving an MRO-based curable system with the polarity modifier isosorbide monomethacrylate (MISD). The illustrative cross-hatch tests for (a) 0 wt % of MISD and (b) 40 wt % of MISD. The coating thickness measurements for all prepared systems and the ISD 2409 adhesion and coating quality rating based on the cross-hatch results.

The wood coating is commonly applied for surface treatment, assuring the hydrophobicity of the polar system, the gloss and color modification of particular products, and the protective character of the substrate. The oil-based wood coatings are often unmodified (pure oils such as castor and linseed oils are used) due to their spontaneous cross-linking in the air oxidizing conditions and increased temperatures.^23^ The curable wood coating systems have advantages such as faster manufacture, property regulations, and more quantitative molecular site formation. **The polarity of the layer-forming system influences the adhesion, durability, and protective properties** since wood is a highly polar system. Various strategies leading to the coat’s surface energy increase are often incorporated, such as silica particle presence^44^ or other polar substance addition (chitosan).^45^ Isosorbide monomethacrylate (MISD) served as an oil-soluble polarity modifier in the article. The cross-hatch (norm ISO 2409) method was selected as an investigation method, and the coating thickness measurements were used to evaluate the coating characteristics. The varying MISD content was set for the wood coating experiments (from 0 wt. up to 40 wt % of MISD in an MRO-based curable system). The demonstrated cross-hatch tests (including the pictures of used tapes), the coating thickness measurements, and the outlined thermoset layer ratings are summarized in Figure 9. The complete cross-hatch pictures are given in the Supporting Information.

We included the illustrative pictures of the edge coating examples (0 and 40 wt % of MISD in MRO-based coating) presented in Figure 9a,b to emphasize the enormous coating quality increase with the most MISD in the layer. The cross-hatch test uncovered the poor damage resistance of 0 wt % of the MISD-containing MRO system, resulting in cracks along the knife cut (see Figure 9a). The adhesion tape test also demonstrated the poor adhesion of unmodified MRO coating due to a solid hydrophobic character. The 0 wt % MISD system reached the level 1 rating, verifying the used curable system’s inferior adhesive and protective properties. On the other hand, the 40 wt % of MISD containing MRO-based mixture coated on the beech wood reached significantly better results. The formed layer knife damage resulted in unobservable cracks along the formed cross, signing the formed coat’s enhanced antidamage characteristics and convenient mechanical properties. Moreover, the tape test proved the exceptional adhesive character of 40 wt % MISD-containing polymer coating since no removed parts were found on the used tape. This outcome assured a level 5 rating for this system, which is the highest obtainable level. The other systems (added in the Supporting Information) demonstrated the apparent improvement of adhesion and the increased damage-protective character of MRO-based coatings with the rising MISD content. Also, the coating thickness reached an average value of 206 ± 12 μm less than a 6% deviation among the prepared samples. This value fulfills the norm’s definition.

### 3.5 Surface Morphology Investigation

**Figure 10:**
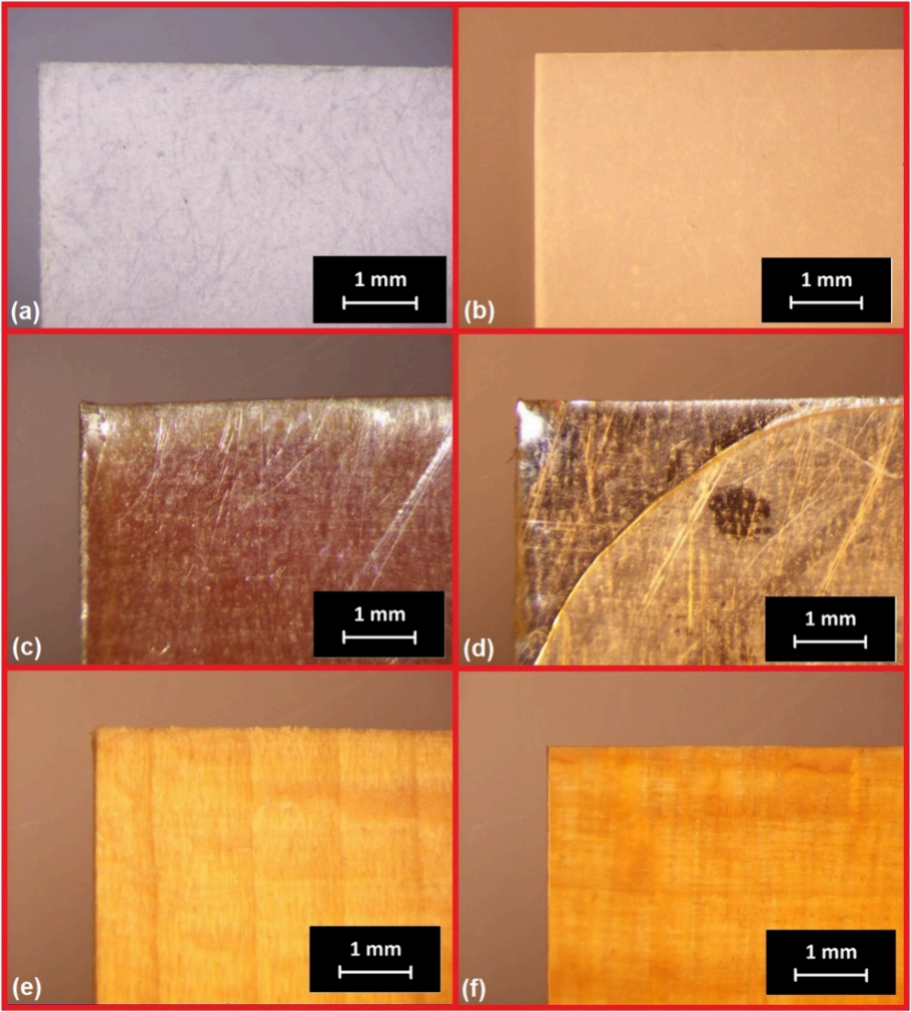
Optical microscopy of all coated substrates. (a) Noncoated paper, (b) coated paper, (c) noncoated stainless steel, (d) coated stainless steel, (e) noncoated beech wood, and (f) coated beech wood.

We performed optical microscopy to investigate noncoated and coated substrates’ morphology and surface character. The obtained images are shown in Figure 10. The paper substrate morphology comparison uncovers the fibrous character of noncoated paper which is exceptionally hydrophilic and the smooth and continual surface after the coating. The coated metal images illustrate the coated shine surface’s mechanical and optical protection. The coated wood exhibits tunable protection against mechanical damage. Also, irradiation protection can proceed when appropriate colorants and additives are added to the thermoset structure due to the continual distribution of the coat.

## 4 Conclusions

This article presents the syntheses and coating applications of curable methacrylated rapeseed oil (MRO), low-viscosity reactive methacrylated alkyl carboxylates (M3HBMMA and E3HBMMA), and water-soluble highly polar isosorbide monomethacrylate (MISD). The productions of named reactive compounds were structurally confirmed via meticulous cross-analysis involving ^1^H NMR, ^13^C NMR, ESI-MS, and FTIR analytical methods. In total, four curable systems were formed to use MRO as a main coating binder for polymer coatings, M3HBMMA and E3HBMMA served as a low-viscous rheology modifier, and MISD’s role was the MRO-based thermoset’s polarity and surface energy increase. The rheological, mechanical, adhesive, and intermolecular properties were investigated via a polymer coating for various substrates, hydrophilic paper, stainless steel, and beech wood. The diluting properties of M3HBMMA were verified based on the paper dip coating results. The layer 87.5% thickness spread for a 100% MRO system decreased to 28.0% measured for a system containing 50 wt % M3HBMMA. Moreover, the additional polymer weight content attached to the paper specimen decreased from 350 to 69 wt %, a significant result for the potential industrial applicability. The paper hydrophobicity was verified via the water contact angle. The flexibility-enhancing factor of E3HBMMA in the MRO-based coating was investigated via metal coating experiments. The bending angle measured for the 100% MRO system reached 12 ± 2°, while the layer containing 40 wt % of E3HBMMA endured the bending to 121 ± 2°. This result confirmed the exceptionally enhanced thermoset coating’s flexibility and adhesion toward the stainless steel substrate. Lastly, the wood coating involved the synthesized polarity modifier, MISD. The surface energy changes affecting the coat’s quality were confirmed since the coating rating based on the norm ISO 2409 reached level 5 (the highest) for the system involving 40 wt % of MISD, while the 100% MRO system obtained a level 1 rating (second worst). Based on the results, the synthesized curable monomers succeeded as a potential reactive coating material precursor.

## Supporting Information Available

Synthesized products’ measured NMR, ESI-MS, and FTIR spectra and complete wood coating documentation (PDF)
