# Supplementary material for: Photocurable Oil-Based Thermosets Containing Modifiers from Renewable Sources for Coating Applications
Source: ACS Polym Au. 2024 Oct 28;4(6):527–39. doi: 10.1021/acspolymersau.4c00068 (PMC11638785; doi:10.1021/acspolymersau.4c00068)
Supplement: Supplementary file 1 — lg4c00068_si_001.pdf [file lg4c00068_si_001.pdf]

Supporting Information

Photo-curable Oil-based Thermosets Containing Modifiers from Renewable Sources for Coating Applications

Vojtěch Jašek[a*], Jan Fučík[b], Otakar Bartoš[a], Silvestr Figalla[a] and Radek Přikryl[a]

a Institute of Materials Chemistry, Faculty of Chemistry, Brno University of Technology, 61200 Brno, Czech Republic.

b Institute of Environmental Chemistry, Faculty of Chemistry, Brno University of Technology, 612 00 Brno, Czech Republic.

*corresponding author: xcjasekv@vutbr.cz

1. Structural characterization of the produced compounds

Methacrylated rapeseed oil (MRO)

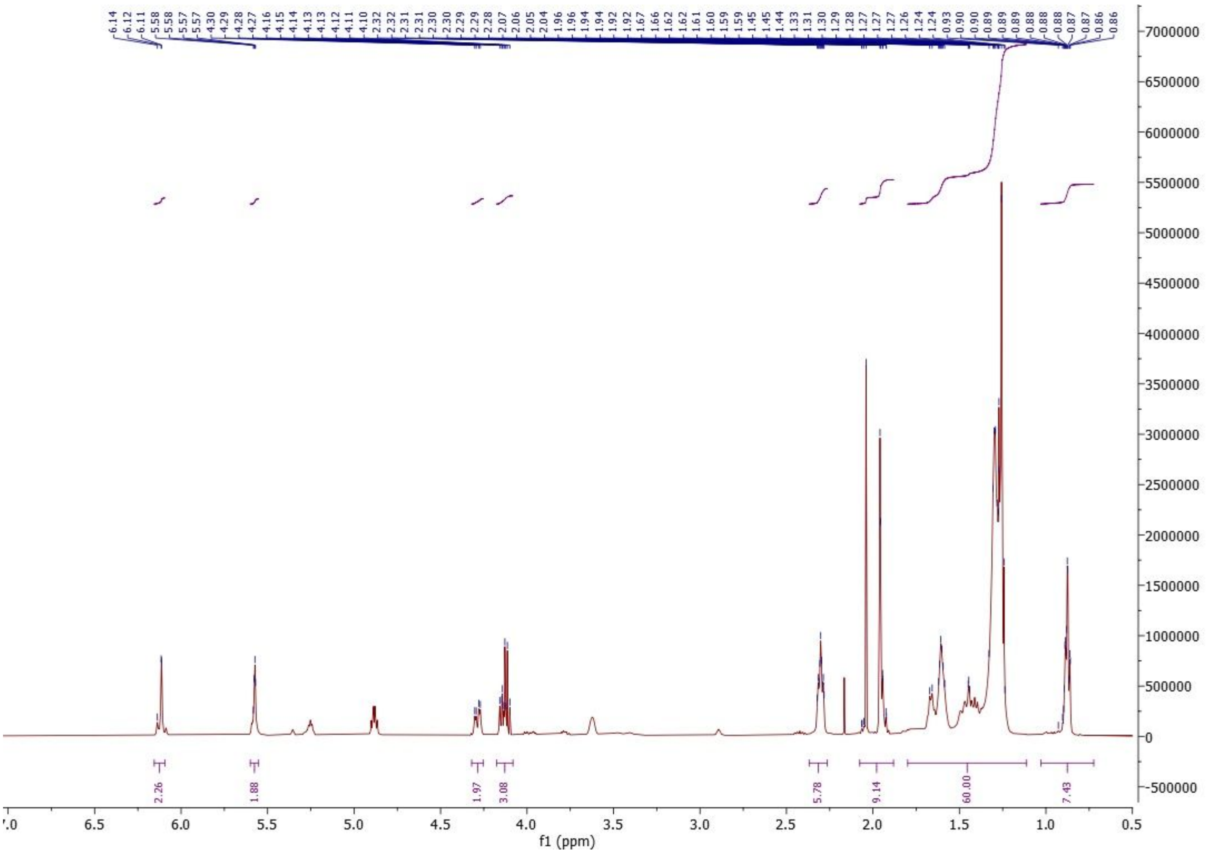

Figure S1. ^1H NMR of methacrylated rapeseed oil (500 MHz, $CDCl^3$): δ 6.16 – 6.09 (m, 2H), 5.58 (q, J = 2.1 Hz, 2H), 4.29 (dd, J = 11.9, 4.3 Hz, 2H), 4.18 – 4.08 (m, 3H), 2.30 (tt, J = 7.5, 2.3 Hz, 6H), 2.08 – 1.88 (m, 9H), 1.80 – 1.11 (m, 60H), 0.88 (tt, J = 7.1, 1.6 Hz, 7H). Reproduced from [14]. Available under CC-BY-4.0 licence. © 2023 by Vojtěch Jašek Et al.

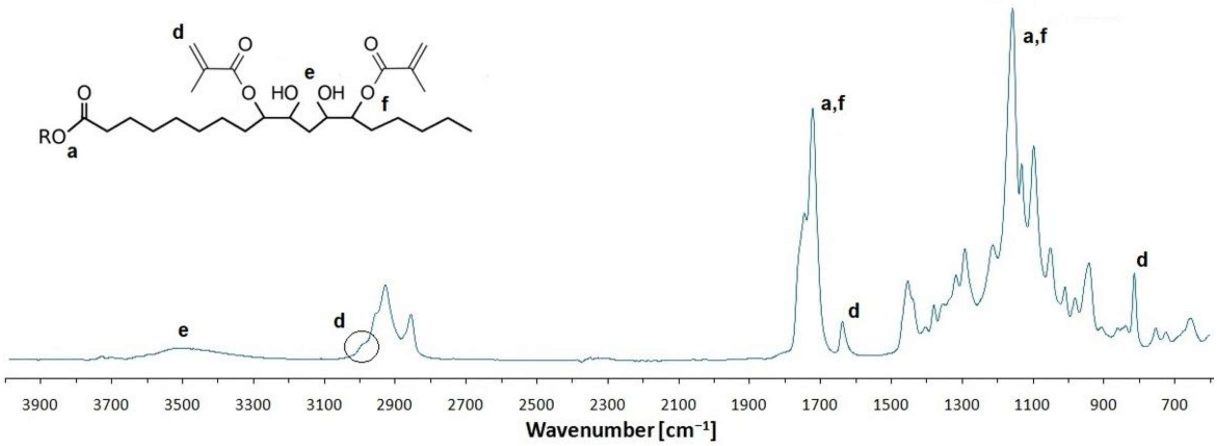

Figure S2. Methacrylated rapeseed oil FTIR spectrum, absorption wavenumber intervals: O-H stretch. 3550-3200 cm^{-1}, C-H stretch. (alkene) 3100-3000 cm^{-1}, C-H stretch. 3000-2840 cm^{-1}, C=O (ester) stretch. 1750-1735 cm^{-1}, C=C stretch. 1662-1626 cm^{-1}, C-O (ester) stretch. 1210-1163 cm^{-1}, C=C bend. 840-790 cm^{-1}. Reproduced from [14]. Available under CC-BY-4.0 licence. © 2023 by Vojtěch Jašek Et al.

Methacrylated alkyl carboxylates (M3HBMMA and E3HBMMA)

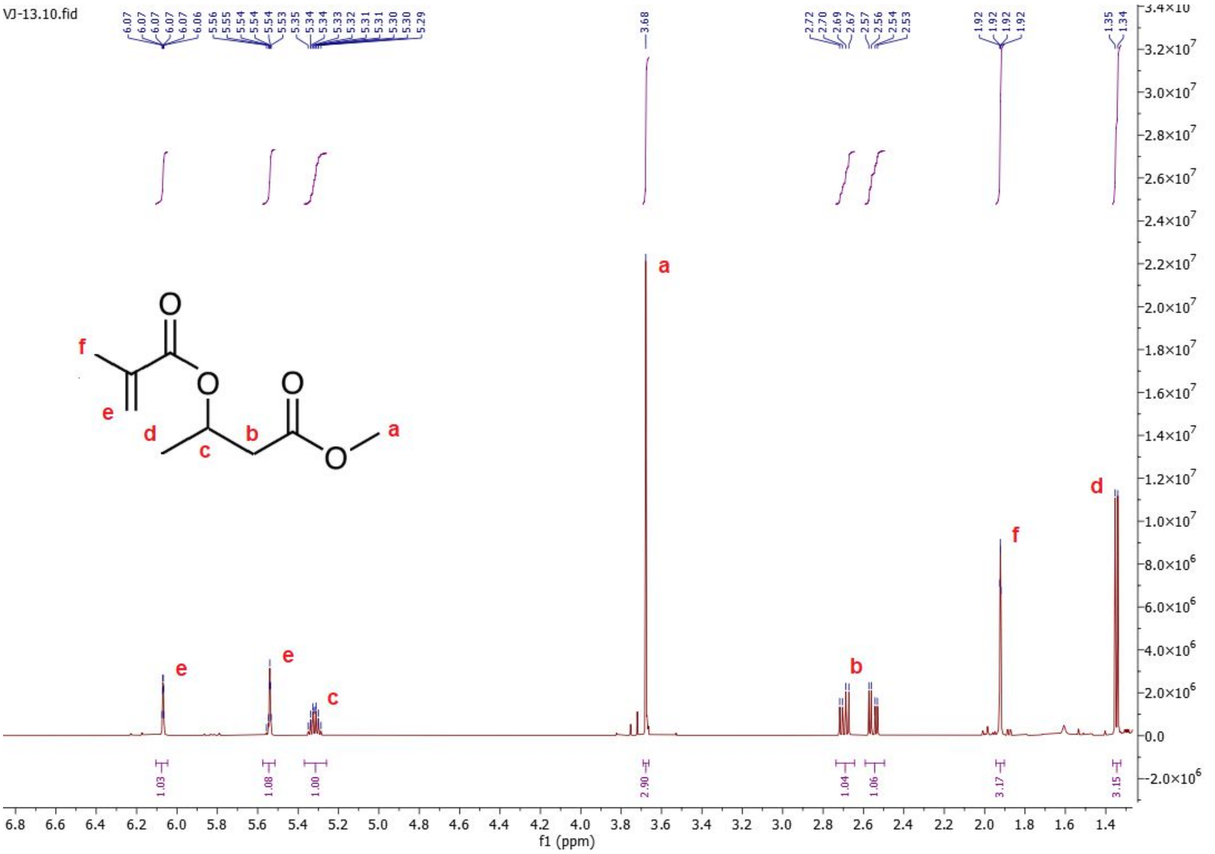

Figure S3. ^{1}H NMR of M3HBMMA ($CDCl_3$, 500 MHz): δ (ppm) 6.07–6.06 (dq; J = 1.96; 1.02; 0.98; 0.98 Hz; 1H), 5.56–5.53 (p; J = 1.60; 1.60; 1.58; 1.58 Hz; 1H), 5.35–5.29 (dp; J = 7.32; 6.26; 6.26; 6.25; 6.25 Hz; 1H), 3.68 (s; 3H), 2.72–2.67 (dd; J = 15.34; 7.29 Hz; 1H), 2.57–2.53 (dd; J = 15.35; 5.79 Hz; 1H), 1.92 (dd; J = 1.63; 1.01 Hz; 3H), 1.35–1.34 (d; J = 6.36 Hz; 3H). Reproduced from [39]. Available under CC-BY-4.0 licence. © 2022 by Vojtěch Jašek Et al.

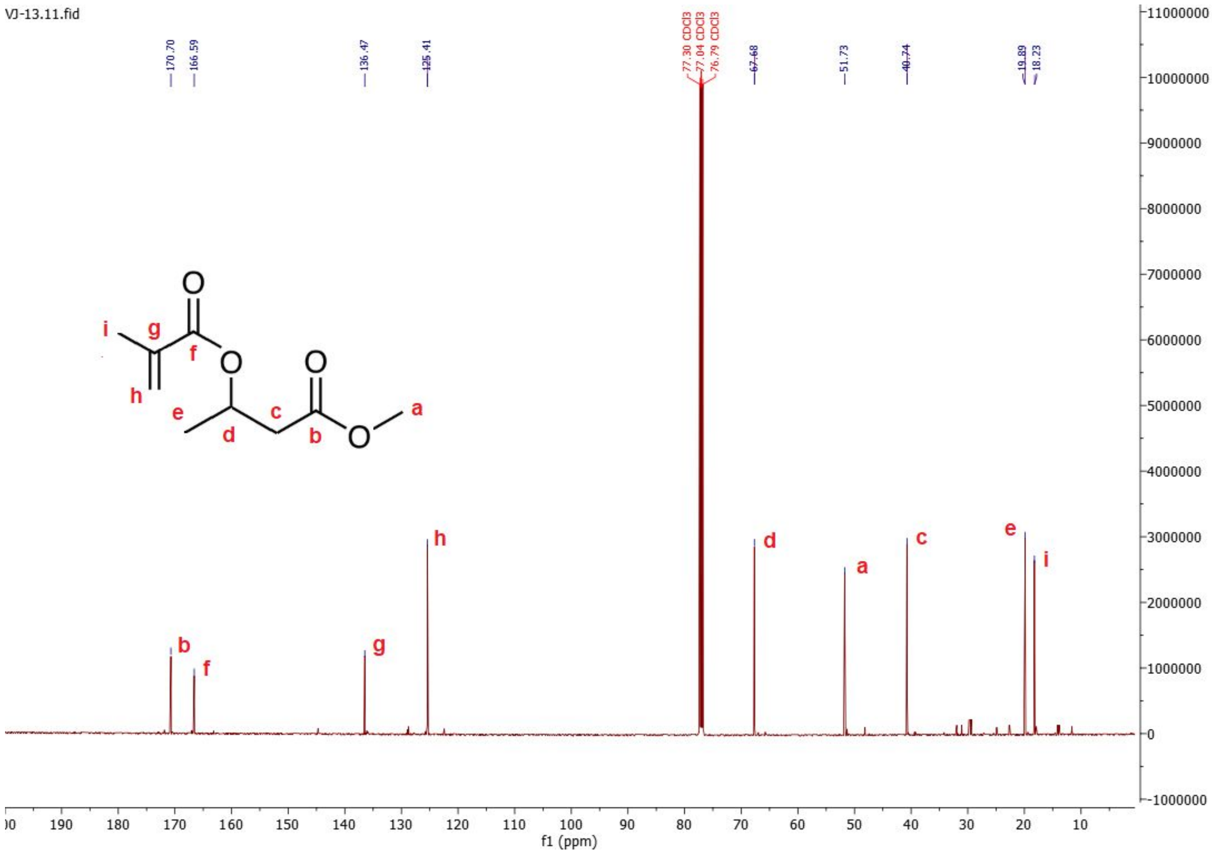

Figure S4. ^{13}C NMR of M3HBMMA ($CDCl_3$, 126MHz): δ (ppm) 170.70; 166.59; 136.47; 125.41; 67.68; 51.73; 40.74; 19.89; 18.23. Reproduced from [39]. Available under CC-BY-4.0 licence. © 2022 by Vojtěch Jašek Et al.

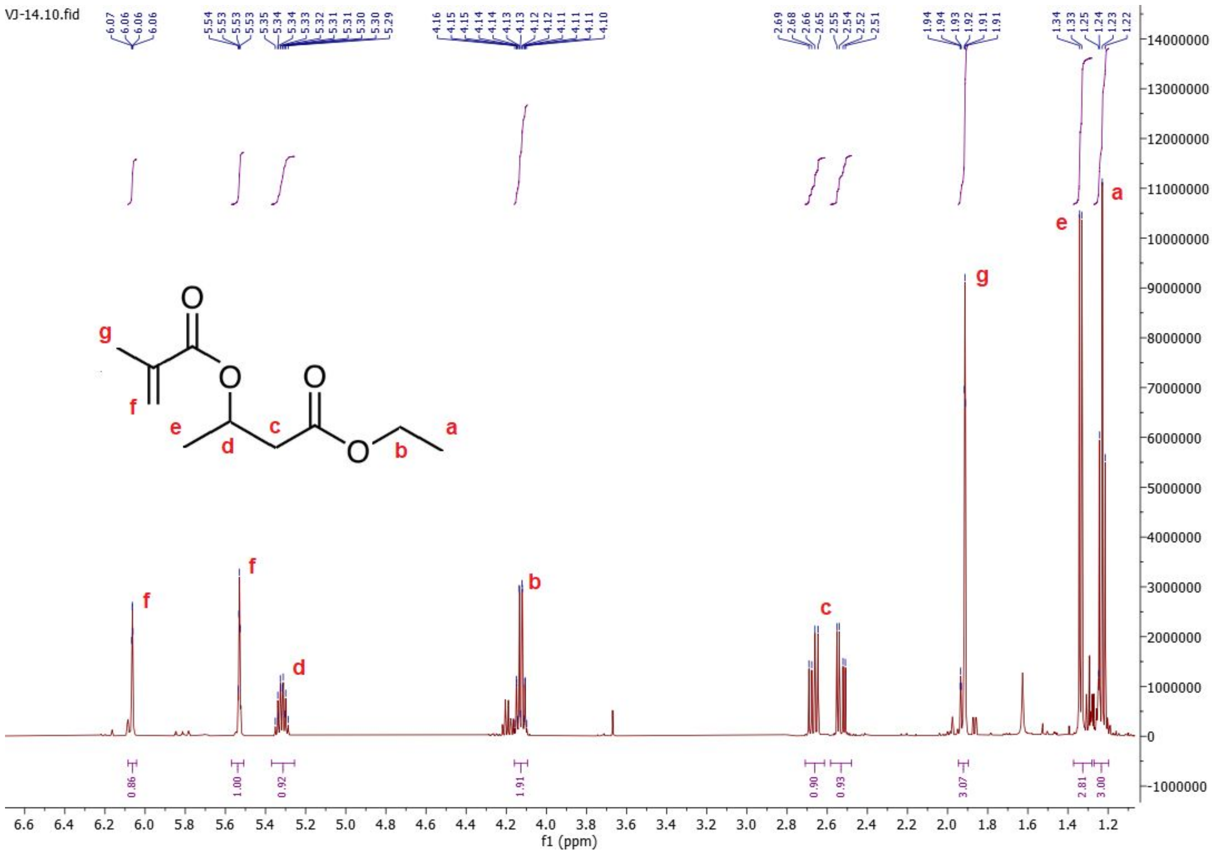

Figure S5. ^{1}H NMR ($CDCl_3$, 500 MHz): δ (ppm) 6.07–6.06 (dd; J = 1.75; 0.97 Hz; 1H), 5.54–5.53 (q; J = 1.63; 1.63; 1.63 Hz; 1H), 5.35–5.29 (dp; J = 7.50; 6.24; 6.24; 6.24; 6.24 Hz; 1H), 4.16–4.10 (qd; J = 7.11; 7.06; 7.06; 0.96Hz; 2H), 2.69–2.65 (dd; J = 15.28; 7.42 Hz; 1H), 2.55–2.51 (dd; J = 15.29; 5.75 Hz; 1H), 1.94–1.91 (m; 3H), 1.34–1.33 (d; J = 6.28 Hz; 3H), 1.25–1.22 (t; J = 7.13; 7.13 Hz; 3H). Reproduced from [39]. Available under CC-BY-4.0 licence. © 2022 by Vojtěch Jašek Et al.

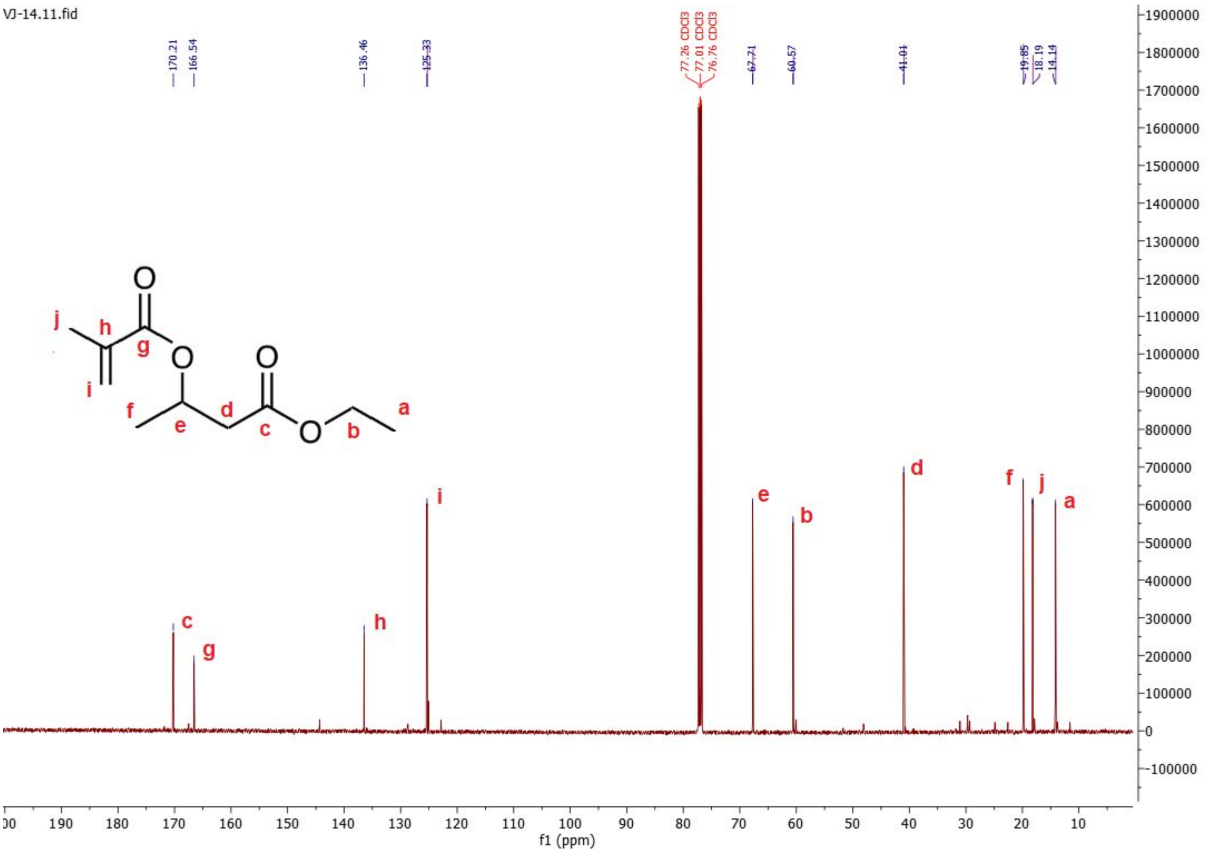

Figure S6. ^{13}C NMR ($CDCl_3$, 126 MHz): δ (ppm) 170.21; 166.54; 136.46; 125.33; 67.71; 60.57; 41.01; 19.85; 18.19; 14.14. Reproduced from [39]. Available under CC-BY-4.0 licence. © 2022 by Vojtěch Jašek Et al.

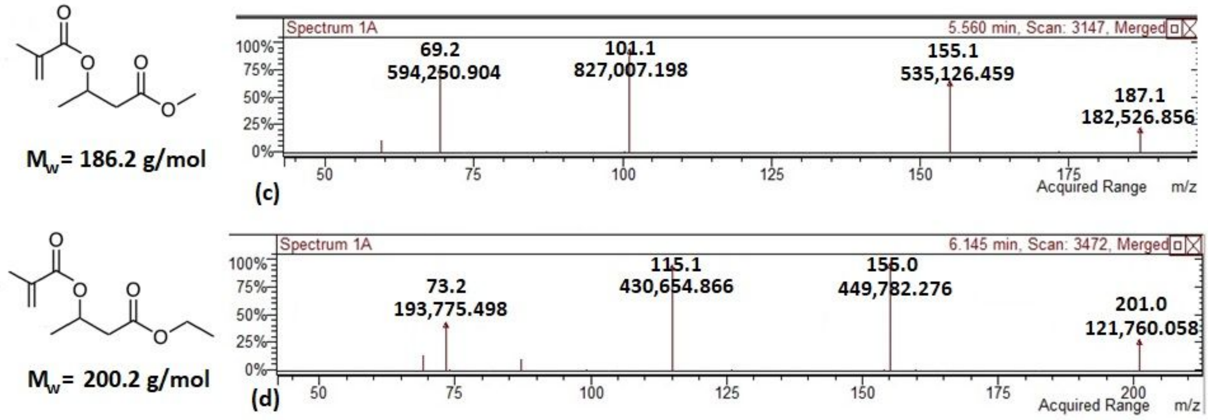

Figure S7. ESI-MS of M3HBMMA analyzed precursor $[M+H]^+$ 187.1 m/z. Fragments: 155.1, 101.1, and 69.2 m/z, and ESI-MS of E3HBMMA analyzed precursor $[M+H]^+$ 201.0 m/z. Fragments: 156.0, 115.1, and 73.2 m/z. Reproduced from [39]. Available under CC-BY-4.0 licence. © 2022 by Vojtěch Jašek Et al.

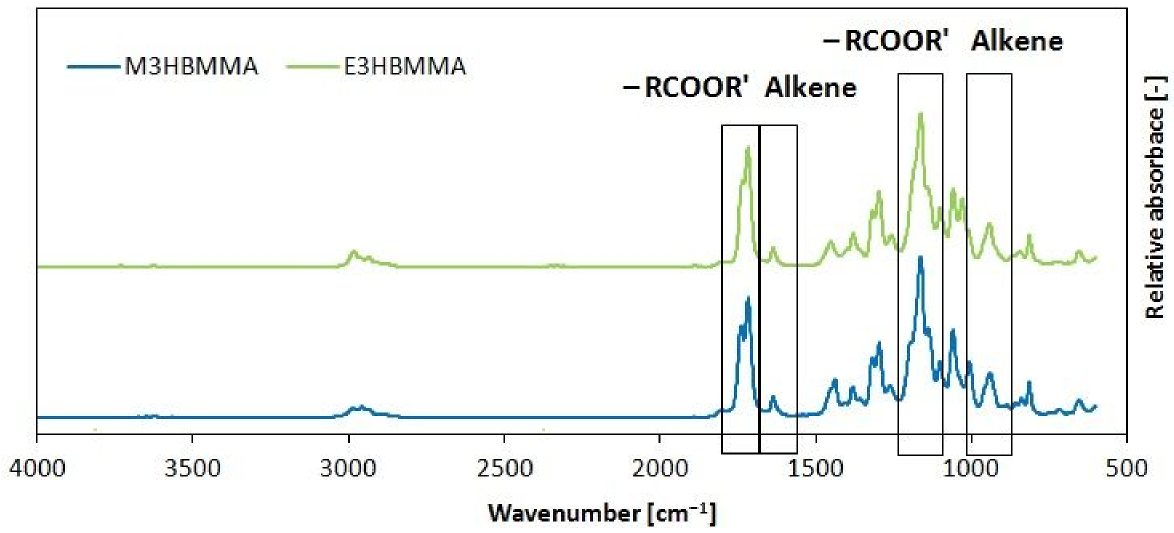

Figure S8. FTIR spectra of M3HBMMA and E3HBMMA, absorption wavenumber intervals: C-H stretch. 3000-2840 cm^{-1}, C=O (ester) stretch. 1750-1735 cm^{-1}, C=C stretch. 1662-1626 cm^{-1}, C-O (ester) stretch. 1210-1163 cm^{-1}, C=C bend. 840-790 cm^{-1}. Reproduced from [39]. Available under CC-BY-4.0 licence. © 2022 by Vojtěch Jašek Et al.

Isosorbide monomethacrylate (MISD)

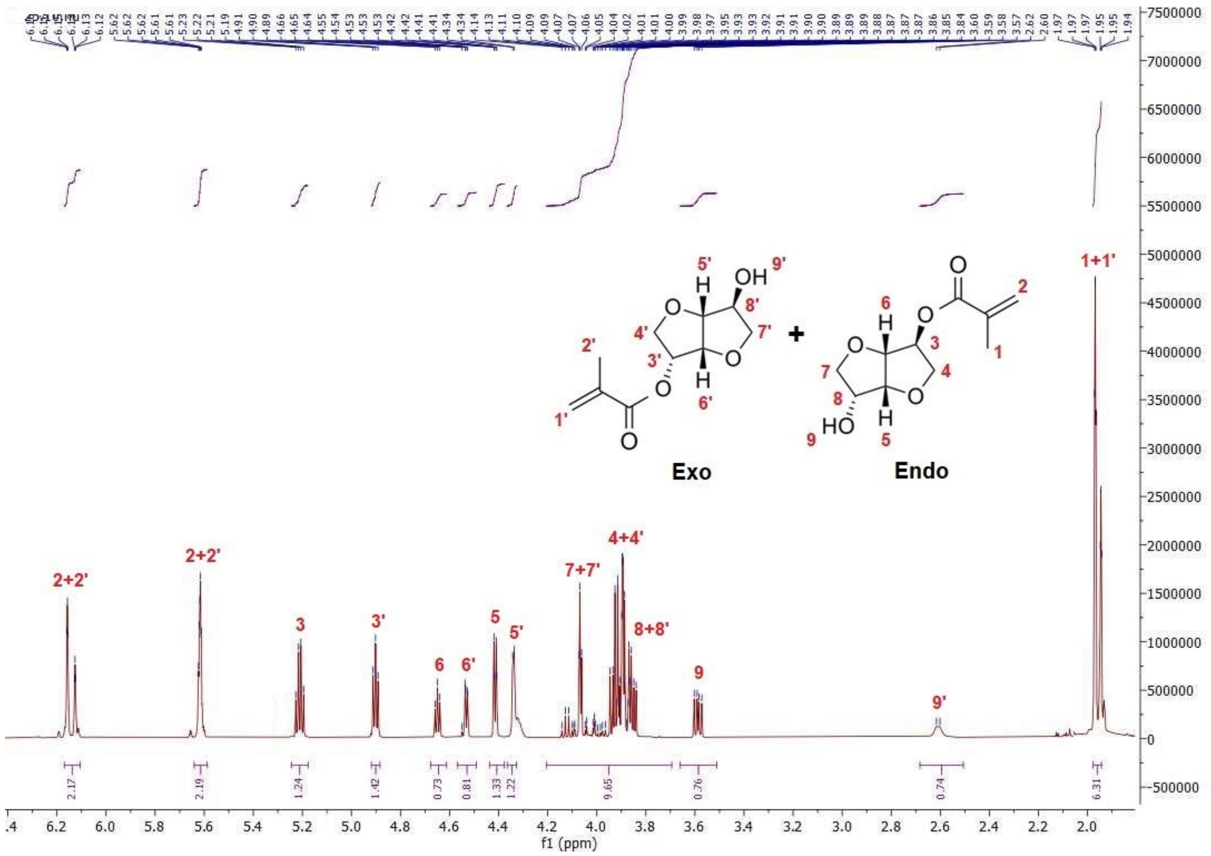

Figure S9. ^{1}H NMR of isosorbide monomethacrylate (MISD) ($CDCl_3$, 500 MHz): δ (ppm) 6.17–6.11 (dt, *J* = 15.6, 1.3 Hz, 2H); 5.64–5.59 (dt, *J* = 3.1, 1.5 Hz, 2H); 5.24–5.18 (m, 1H); 4.92–4.88 (t, *J* = 5.1 Hz, 1H); 4.68–4.61 (t, *J* = 4.8 Hz, 1H); 4.57–4.49 (dd, *J* = 4.4, 1.2 Hz, 1H); 4.44–4.38 (dd, *J* = 4.6, 1.1 Hz, 1H); 4.37–3.33 (d, *J* = 3.2 Hz, 1H); 4.21–3.69 (m, 10H); 3.66–3.51 (dd, *J* = 9.5, 6.0 Hz, 1H); 2.68–2.51 (d, *J* = 7.0 Hz, 1H); 1.98–1.94 (m, 6H). Reproduced from [40]. Available under CC-BY-4.0 licence. © 2024 by Vojtěch Jašek Et al.

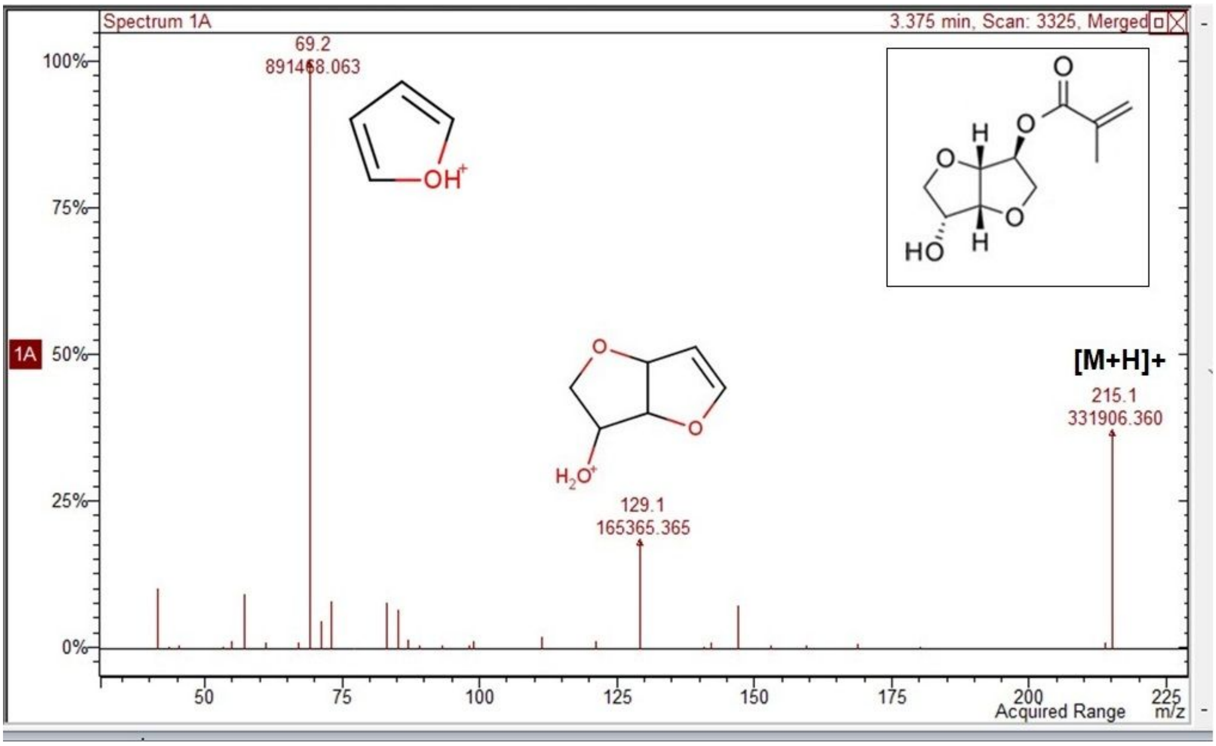

Figure S10. ESI-MS of isosorbide monomethacrylate (MISD) analyzed precursor $[M+H]^+$ 215.1 m/z. Fragments: 129.1 and 62.9 m/z. Reproduced from [40]. Available under CC-BY-4.0 licence. © 2024 by Vojtěch Jašek Et al.

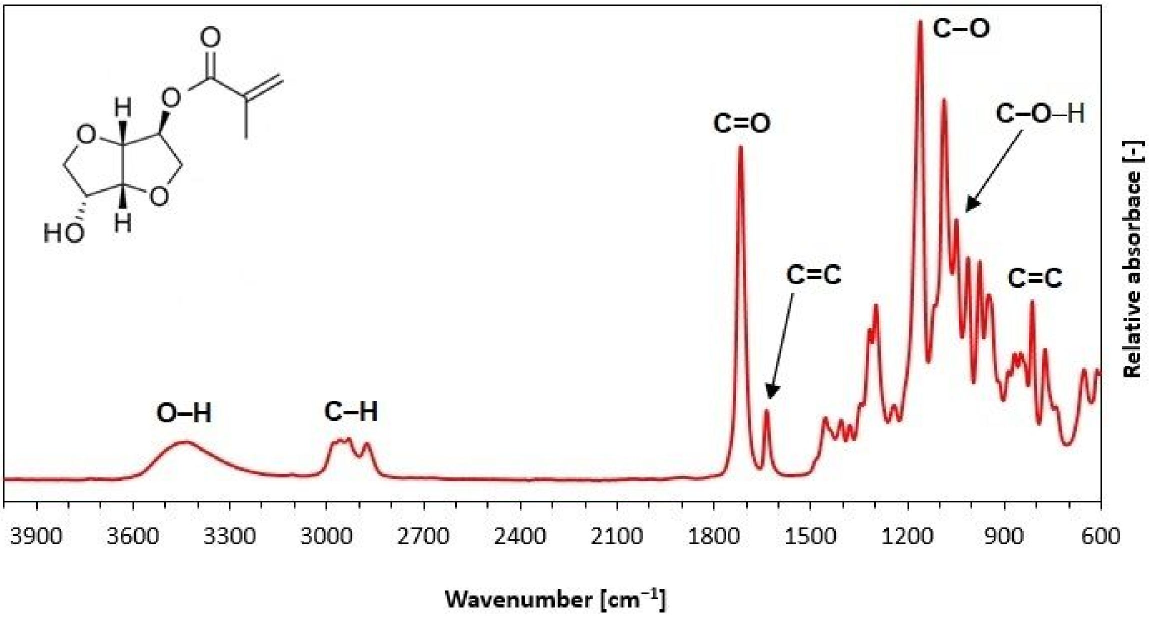

Figure S11. FTIR spectrum of isosorbide monomethacrylate (MISD), absorption wavenumber intervals: O-H stretch. 3550-3200 cm^{-1}, C-H stretch. 3000-2840 cm^{-1}, C=O (ester) stretch. 1750-1735 cm^{-1}, C=C stretch. 1662-1626 cm^{-1}, C-O (ester and alcohol) stretch. 1210-1163 cm^{-1}, C=C bend. 840-790 cm^{-1}. Reproduced from [40]. Available under CC-BY-4.0 licence. © 2024 by Vojtěch Jašek Et al.

2. Additional data on the wood coating

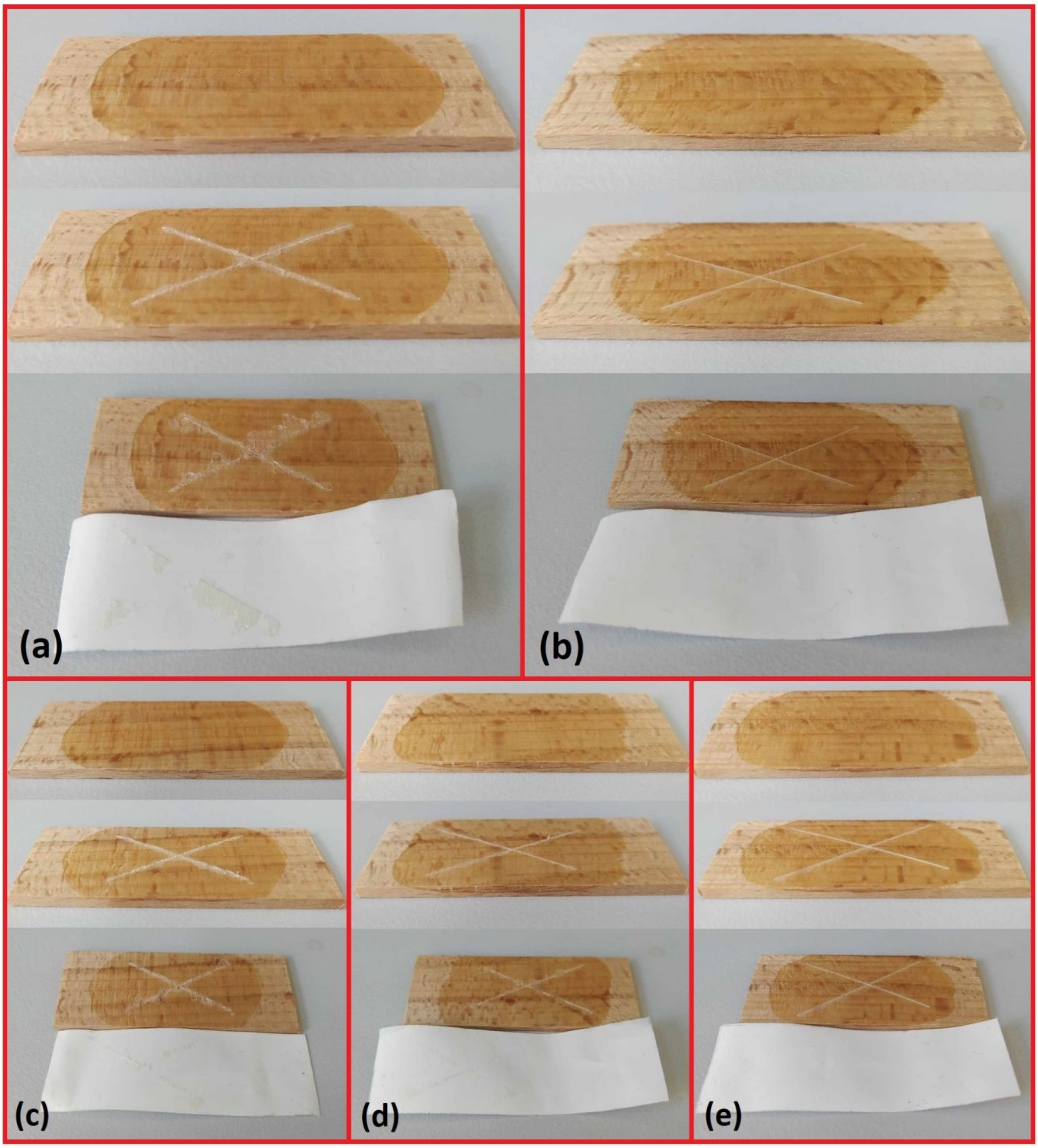

Figure S12. The wood coating performed with MRO-based MISD-containing curable mixtures. (a) 0 wt.% of MISD, (b) 40 wt.% of MISD, (c) 10 wt.% of MISD, (d) 20 wt.% of MISD, (e) 30 wt. % of MISD.
